# Clathrin- and dynamin-dependent endocytosis limits canonical NF-κB signaling triggered by lymphotoxin β receptor

**DOI:** 10.1186/s12964-020-00664-0

**Published:** 2020-11-04

**Authors:** Małgorzata Maksymowicz, Marta Miączyńska, Magdalena Banach-Orłowska

**Affiliations:** Laboratory of Cell Biology, International Institute of Molecular and Cell Biology, 02-109 Warsaw, Poland

**Keywords:** Endocytosis, Lymphotoxin β receptor, NF-κB signaling, Receptor internalization, Dynamin, Clathrin-mediated endocytosis, Clathrin-independent endocytosis

## Abstract

**Background:**

Lymphotoxin β receptor (LTβR) is a member of tumor necrosis factor receptor (TNFR) superfamily which regulates the immune response. At the cellular level, upon ligand binding, the receptor activates the pro-inflammatory NF-κB and AP-1 pathways. Yet, the intracellular distribution of LTβR, the routes of its endocytosis and their connection to the signaling activation are not characterized. Here, we investigated the contribution of LTβR internalization to its signaling potential.

**Methods:**

Intracellular localization of LTβR in unstimulated and stimulated cells was analyzed by confocal microscopy. Endocytosis impairment was achieved through siRNA- or CRISPR/Cas9-mediated depletion, or chemical inhibition of proteins regulating endocytic routes. The activation of LTβR-induced signaling was examined. The levels of effector proteins of the canonical and non-canonical branches of the NF-κB pathway, and the phosphorylation of JNK, Akt, ERK1/2, STAT1 and STAT3 involved in diverse signaling cascades, were measured by Western blotting. A transcriptional response to LTβR stimulation was assessed by qRT-PCR analysis.

**Results:**

We demonstrated that LTβR was predominantly present on endocytic vesicles and the Golgi apparatus. The ligand-bound pool of the receptor localized to endosomes and was trafficked towards lysosomes for degradation. Depletion of regulators of different endocytic routes (clathrin-mediated, dynamin-dependent or clathrin-independent) resulted in the impairment of LTβR internalization, indicating that this receptor uses multiple entry pathways. Cells deprived of clathrin and dynamins exhibited enhanced activation of canonical NF-κB signaling represented by increased degradation of IκBα inhibitor and elevated expression of LTβR target genes. We also demonstrated that clathrin and dynamin deficiency reduced to some extent LTβR-triggered activation of the non-canonical branch of the NF-κB pathway.

**Conclusions:**

Our work shows that the impairment of clathrin- and dynamin-dependent internalization amplifies a cellular response to LTβR stimulation. We postulate that receptor internalization restricts responsiveness of the cell to subthreshold stimuli.

**Video Abstract**

**Graphical abstract:**

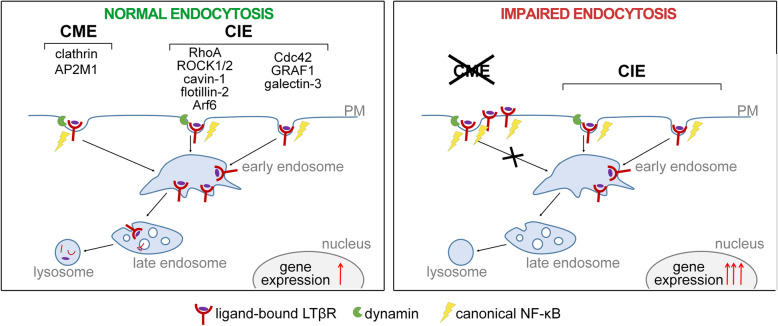

**Supplementary information:**

**Supplementary information** accompanies this paper at 10.1186/s12964-020-00664-0.

## Background

Lymphotoxin β receptor (LTβR), a member of tumor necrosis factor receptor superfamily (TNFRSF), is a transmembrane cytokine receptor. It controls the development and maintenance of secondary lymphoid organs: lymph nodes [1] and Peyer’s patches [2], and immune cell development [3–6]. LTβR is constitutively and ubiquitously expressed, with an exception of T and B lymphocytes [7, 8], which in turn are the source of its two ligands: lymphotoxin α1β2 (LTα1β2) and LIGHT [9, 10].

Ligand binding by LTβR activates signaling cascades, leading to NF-κB- and AP-1-dependent transcription [10–15]. The best characterized in this context is NF-κB signaling, operating via two branches: the canonical and non-canonical ones [11, 12, 15]. Upon stimulation, LTβR binds TNFR-associated factor (TRAF) adaptors, that in the canonical branch is followed by proteasomal degradation of a pathway inhibitor, IκBα. This enables RelA/p50 heterodimer release and translocation to the nucleus. In the non-canonical branch TRAF binding is followed by accumulation of NF-κB-inducing kinase (NIK), leading to the degradation of an inhibitory domain of p100. The resulting transcriptionally active p52 form, together with RelB, acts as a transcription factor (reviewed in [16]). In consequence, activation of both branches induces expression of NF-κB target genes [11, 12, 15, 16]. LTβR-ligand binding also activates a signaling cascade which operates through AP-1 transcription factors. Within this pathway, the action of mitogen-activated protein kinases (MAPKs), including JNK and ERK1/2, activates c-Jun or ATF2, which together with other proteins (e.g. cFos, JunB) form AP-1 transcription factors [13, 17, 18]. LTβR was also reported to regulate the activity of diverse transcription factors, such as STAT1 [19] and STAT3 [20].

Numerous studies show that a particular receptor can be endocytosed via multiple pathways [21–23]. There is a large number of endocytic routes, which are classically divided into clathrin-mediated endocytosis (CME) and clathrin-independent endocytosis (CIE). In CME, cargo is internalized via clathrin-coated pits due to interactions with adaptor proteins. One of them is the adaptor protein 2 (AP2) complex consisting of: α, β2, μ2 and σ2 subunits, among which the μ2 subunit (encoded by *AP2M1* gene) is essential for cargo recognition [24, 25]. A nascent clathrin-coated vesicle is cut off from the plasma membrane (PM) by a large GTPase, dynamin [26–28]. There are three dynamin proteins in mammalian cells: dynamin-1, expressed mostly in neurons, dynamin-2, which is ubiquitously expressed, and dynamin-3, expressed in neurons, testes and lungs [29, 30]. It was shown that some cancer cell lines express both dynamin-1 and dynamin-2, which play complementary roles in the regulation of endocytosis [27, 31–33]. Dynamins can also be involved in some forms of CIE [34].

CIE is a general term for various endocytic routes, distinguished based on several criteria: the dependence on dynamin, the type of coating proteins (e.g. caveolins, flotillins) or involvement of small GTPases (e.g. Arf6, Cdc42, RhoA). One example of CIE is a caveolae-dependent pathway. It is regulated by dynamin and operates through caveolae, which are formed by caveolins and cavins [35]. We recently found that LTβR internalization depends on cavin-1 [36]. Another CIE route relying on the dynamin activity is RhoA-dependent endocytosis, which is regulated by RhoA together with its effectors, Rho-associated kinases: ROCK1 and ROCK2 [37–39]. In turn, endocytosis involving clathrin-independent carriers (CLIC)/glycosylphosphotidylinositol-anchored protein (GPI-AP) enriched compartments (GEEC), regulated by small GTPase Cdc42, and GTPase activating protein GRAF1, does not require dynamin [40–42]. Moreover, there are routes which involve flotillins [43, 44], Arf6 [34, 45, 46] and curvature-generating/sensing membrane protein endophilin. The latter operates both in CME and in one of CIE routes termed fast endophilin mediated endocytosis (FEME) [47].

Endocytosis regulates receptor signaling in different ways. Firstly, internalization decreases the amounts of receptors available on the PM for binding extracellular ligands. Secondly, internalized receptors can be transported via endocytic routes towards degradation or recycled back to the PM that modulates the kinetics and strength of signal transmission [27, 48–51]. Finally, some receptors propagate signals from endosomes, which play a role of intracellular signaling platforms [52, 53]. While there are many reports on tyrosine kinase receptors (RTKs) and G protein-coupled receptors (GPCRs), showing the link between endocytosis and signaling [23, 54, 55], the knowledge on such regulation for cytokine receptors is limited [22]. Most of the studies on LTβR focus on its role in development and maintenance of immune system [56, 57], as well as its contribution to the progress of different diseases [10, 58]. In contrast, there are only a few reports on the cell biology of LTβR.

In our previous research we showed that blocking intracellular trafficking led to the accumulation of ligand-free LTβR on endosomes. Depending on the topology of the receptor in endocytic vesicles, it strongly activated NF-κB signaling in a ligand-independent manner (when the receptor was localized on the outer endosomal membrane) [59] or had no effect on signaling (when the receptor was inside intraluminal vesicles of endosomes) [60]. Another study revealed that LTβR-driven activation of the non-canonical NF-κB pathway required dynamin-2-dependent endocytosis [61].

Internalization, trafficking, intracellular distribution of LTβR, as well as the link between endocytosis and signaling of LTβR are still poorly described. In this study we addressed all these issues. We characterized the cellular distribution of the receptor, identified its multiple internalization routes and found clathrin- and dynamin-dependent endocytosis important for the activation of canonical NF-κB signaling.

## Methods

### Cell culture and treatments

A549 pulmonary adenocarcinoma cells (ECACC, #86012804) were purchased from Sigma-Aldrich. HEK293T, CCD1070Sk and HeLa cell lines were from ATCC. A549 and HEK293T cells were maintained in Dulbecco’s Modified Eagle’s Medium (DMEM) high glucose, supplemented with 10% fetal bovine serum (FBS) and 2 mM L-glutamine (all purchased from Sigma-Aldrich). HeLa and CCD1070Sk cells were maintained in Modified Eagle’s Medium (MEM) high glucose, supplemented with 10% fetal bovine serum (FBS) and 2 mM L-glutamine. Mycoplasma contamination tests were routinely performed.

For stimulation and other non-transfection-based experiments, cells were seeded on a 12-well plate (1–2.4x10^5^cells/well) for Western blotting and quantitative real time PCR (qRT-PCR) experiments, or on 12 mm coverslips in a 24-well plate (5 × 10^4^ cells/well) for microscopy, then treated and lysed or fixed the following day. LTβR stimulation was performed with anti-LTβR agonistic antibody, Ago (R&D Systems, #AF629), Recombinant Human Lymphotoxin α1β2 Protein (R&D Systems, #8884-LY) and Recombinant Human LIGHT/TNFSF14 Protein (R&D Systems, #AF664), all used at concentration 0.2 μg/ml. Transferrin-Alexa-Fluor-647 (Thermo Fisher Scientific, #T23366) was added for 30 min at concentration 25 μg/ml during stimulation.

The following chemical inhibitors were used to block endocytic routes: dynasore (100 μM, 1 h pretreatment; #HY-15304), chlorpromazine hydrochloride (20 μM, 0.5 h pretreatment; #HY-B0407A) from MedChemExpress, ML141 (10 μM, 1.5 h pretreatment; #SML0407) from Sigma-Aldrich. To inhibit lysosomal degradation, cells were treated with chloroquine (CQ, #C6628, Sigma-Aldrich). The concentrations of CQ used in experiments were as follows: 100 μM for HeLa, 64 μM for A549 and HEK293T and 32 μM for CCD1070Sk cells. After 16 h (HeLa) or 20 h (A549, HEK293T and CCD1070Sk) of incubation with CQ or vehicle, cells were additionally stimulated or not for 4 h with Ago.

All experiments were performed using cells cultured in full medium (DMEM and MEM supplemented with 10% FBS and 2 mM L-glutamine), except those employing inhibitors of endocytosis and transferrin uptake, where starvation medium (DMEM and MEM supplemented with 2 mM L-glutamine) was applied.

### Antibodies and chemicals

The list of primary antibodies is presented in Additional file 1: Table 1.

Secondary antibodies used for Western blotting, diluted 1:10,000, were as follows: horseradish peroxidase (HRP)-conjugated anti-goat, anti-mouse and anti-rabbit (Jackson ImmunoResearch Labs, #805–035-180, #115–035-062, #111–035-144, respectively); secondary fluorophore-conjugated anti-mouse IRDye 800CW and anti-rabbit IRDye 680CW (LI-COR Biosciences, #926–32,212 and #926–68,023, respectively).

For immunofluorescence, the following secondary antibodies were used: Alexa Fluor 488-, 555-, 647-conjugated anti-goat, anti-mouse and anti-rabbit, purchased from Thermo Fisher Scientific, diluted 1:500. Phalloidin-Atto-390 (#50556; 1:1000), used for actin staining, was from Sigma-Aldrich.

### Transfection with small interfering RNA (siRNAs)

Reverse transfections with siRNAs were performed with Lipofectamine® RNAiMAX transfection reagent (Thermo Fisher Scientific) according to the manufacturer’s instructions. A549, HEK293T and CCD1070Sk cells were seeded on a 12-well plate (6-16 × 10^4^ cells/well) for Western blotting and qRT-PCR experiments or on 12 mm coverslips in a 24-well plate (3 × 10^4^ cells/well) for microscopy (A549 cells only). Cells were analyzed 72 h post transfection. If cells were stimulated, the ligand was applied for certain time periods prior to lysis or fixation.

We used the following Ambion Silencer Select siRNAs (Thermo Fisher Scientific): Ctrl 1 (Negative Control No. 1, 4,390,843), Ctrl 2 (Negative Control No. 2, 4,390,846), Arf6 1 (s1565; GUCUCAUCUUCGUAGUGGAtt), Arf6 2 (s1566; AGACGGUGACUUACAAAAAtt), Arf6 3 (s1567; CCAAGGUCUCAUCUUCGUAtt), Cdc42 1 (s2765; UGGUGCUGUUGGUAAAACAtt), Cdc42 2 (s2767; CAGUUAUGAUUGGUGGAGAtt), CHC 1 (s475; GGUUGCUCUUGUUACGGAUtt), CHC 2 (s477; GGGAAUUCUUCGUACUCCAtt), Dynamin-1 1 (s144; GCAGUUCGCCGUAGACUUUtt), Dynamin-1 2 (s146; GCACUGCAAGGGAAAGAAAtt), Dynamin-2 1 (s4212; ACAUCAACACGAACCAUGAtt), Dynamin-2 2 (s4214; AGCGAAUCGUCACCACUUAtt), Endophilin-A2 1 (s12796; GCAAGGCGGUGACAGAAGUtt), Endophilin-A2 2 (s12797; GACUUUGACUACAAGAAGAtt), Endophilin-A2 3 (s12798; GCUUCAUCGUCUUUCCGAUtt), Flotillin-1 1 (s19913; GCAGAGAAGUCCCAACUAAtt), Flotillin-1 2 (s19914; GCAUCAGUGUGGUUAGCUAtt), Flotillin-1 3 (s19915; AGAGAGAUUACGAACUGAAtt), Flotillin-2 1 (s5284; CCAAGAUUGCUGACUCUAAtt), Flotillin-2 2 (s5285; ACAGUAAGGUCACAUCAGAtt), Flotillin-2 3 (s5286; GACUAUAAACAGUACGUGUtt), Galectin-3 1 (s8148; GGAGAGUCAUUGUUUGCAAtt), Galectin-3 2 (s8149; CGGUGAAGCCCAAUGCAAAtt), Galectin-3 3 (s8150; GACAGUCGGUUUUCCCAUUtt), GRAF1 1 (s23013; GAGCAAGGGCUGUAUCGAAtt), GRAF1 2 (s23015; GGAUACGGAUGAUUGAGAAtt), RhoA 1 (s759; CUAUGAUUAUUAACGAUGUtt), RhoA 2 (s760; GGCUUUACUCCGUAACAGAtt), ROCK1 1 (s12097; GGUUAGAACAAGAGGUAAAtt), ROCK1 2 (s12098; CGGUUAGAACAAGAGGUAAtt), ROCK2 1 (s18162; GAGAUUACCUUACGGAAAAtt), ROCK2 2 (s18161; GGAGAUUACCUUACGGAAAtt).

All siRNAs were used at concentration of 20 nM, except double dynamin-1/2 knock-down where each siRNA was used at concentration of 15 nM. In case of double dynamin-1/2 silencing, the following combinations of siRNAs were used: dynamin-1/2 1 (Dynamin-1 1 & Dynamin-2 1), dynamin-1/2 2 (Dynamin-1 2 & Dynamin-2 1), dynamin-1/2 3 (Dynamin-1 1 & Dynamin-2 2). For these experiments non-targeting siRNAs were combined as follows: Ctrl 1 (15 nM of Ctrl 1 and 15 nM of Ctrl 2) and Ctrl 2 (30 nM of Ctrl 2).

### Immunofluorescence and image quantification

Upon treatment, A549 cells grown on coverslips were transferred to ice and washed with ice-cold PBS, then fixed with 3.6% paraformaldehyde in PBS for 15 min at room temperature and kept in PBS at 4 °C to be later immunostained, as described previously [59, 62].

To stain exclusively the ligand-bound pool of LTβR, cells stimulated with Ago were incubated without the primary LTβR antibody. Instead, only secondary anti-goat antibody was used to specifically stain Ago-LTβR complexes [36]. Images were acquired with Zeiss LSM 710 confocal microscope using 40×/1.30 oil immersion objective and 1024 × 1024 pixel resolution, obtaining 10 images per each experimental condition. Images were imported to MotionTracking software (http://motiontracking.mpi-cbg.de) which measures integral intensity (reflecting the amounts of a protein on vesicular structures) and number of vesicles as well as colocalization of stained proteins [63–65]. Figures were assembled in Photoshop (Adobe) with only linear adjustments of contrast and brightness.

### Western blot analysis

Cells were transferred to ice and washed with ice-cold PBS, then lysed in RIPA lysis buffer (1% Triton X-100, 0.5% sodium deoxycholate, 0.1% SDS, 50 mM Tris pH 7.4, 150 mM NaCl, 0.5 mM EDTA) in the presence of protease inhibitors (6 μg/ml chymostatin, 0.5 μg/ml leupeptin, 10 μg/ml antipain, 2 μg/ml aprotinin, 0.7 μg/ml pepstatin A and 10 μg/ml 4-amidinophenylmethanesulfonyl fluoride hydrochloride, Sigma-Aldrich) and phosphatase inhibitors (P0044 and P5726, Sigma-Aldrich).

Protein concentration was measured using BCA Protein Assay Kit (Thermo Fisher Scientific). Lysates of 20–30 μg protein were boiled in Laemmli sample buffer for 10 min at 95 °C and then resolved on 10–12% polyacrylamide gels, followed by transfer onto nitrocellulose membrane (GE Healthcare). Membranes were first blocked in 5% milk in PBS-T or 5% BSA in TBS-T, then incubated overnight with specific primary, and for 1 h with secondary antibodies. To detect the signal, ChemiDoc system (Bio-Rad) or Odyssey infrared imaging system (LI-COR Biosciences) were used. Densitometry analysis of detected bands was performed with ImageJ Software [66].

### Quantitative real-time PCR (qRT-PCR)

Total RNA was isolated using High Pure RNA Isolation Kit (Roche) in accordance with the manufacturer’s protocol. cDNA synthesis was performed using M-MLV reverse transcriptase, random nonamers and oligo (dT)_23_ from Sigma-Aldrich, according to the manufacturer’s instructions.

The primers used for qRT-PCR are listed in Additional file 1: Table 2. Reactions were performed with KAPA SYBR FAST qPCR Master Mix (2X) Universal Kit (KK4618, KapaBiosystems), using a 7900HT Fast Real-Time PCR System (Applied Biosystems). Data analysis was performed in Data Assist v3.01 software (Applied Biosystems). The expression of target genes was normalized to the level of transcripts of *ACTB* and *B2M* housekeeping genes, and presented as fold changes.

### Generation of AP2M1 knock-out A549 cell lines by CRISPR/Cas9 gene editing

Knock-out of AP2M1 was performed in A549 cells, using CRISPR/Cas9 gene editing technology as described [36, 60]. Two 25-bp-long single guide RNA (sgRNAs) were designed using the Brunello library [67] (sequences listed in Additional file 1: Table 3), followed by cloning into LentiCRISPR v2 vector (Addgene #52961). Additionally, plasmids containing non-targeting sgRNA (kind gift from Dr. Katarzyna Mleczko-Sanecka) were designed as described [68].

Production of lentiviruses, infection of A549 cells, and clonal selection were performed according to the protocol described before [36, 60]. For each sgRNA targeting AP2M1, one clone was selected, based on the best knock-down efficiency. For non-targeting sgRNA four clones were selected and pooled.

### Statistical analyses

Each experiment was done in at least three repetitions. Data were analyzed in Prism 6 (GraphPad Software). To check the significance of differences in fold changes vs control (set as 1), one sample *t* test was used. The significance of mean comparison is annotated as follows: non-significant, ns - *P* > 0.05, *P ≤ 0.05, **P ≤ 0.01 and ***P ≤ 0.001.

## Results

### LTβR localizes to endosomes and Golgi apparatus

**Fig. 1:**
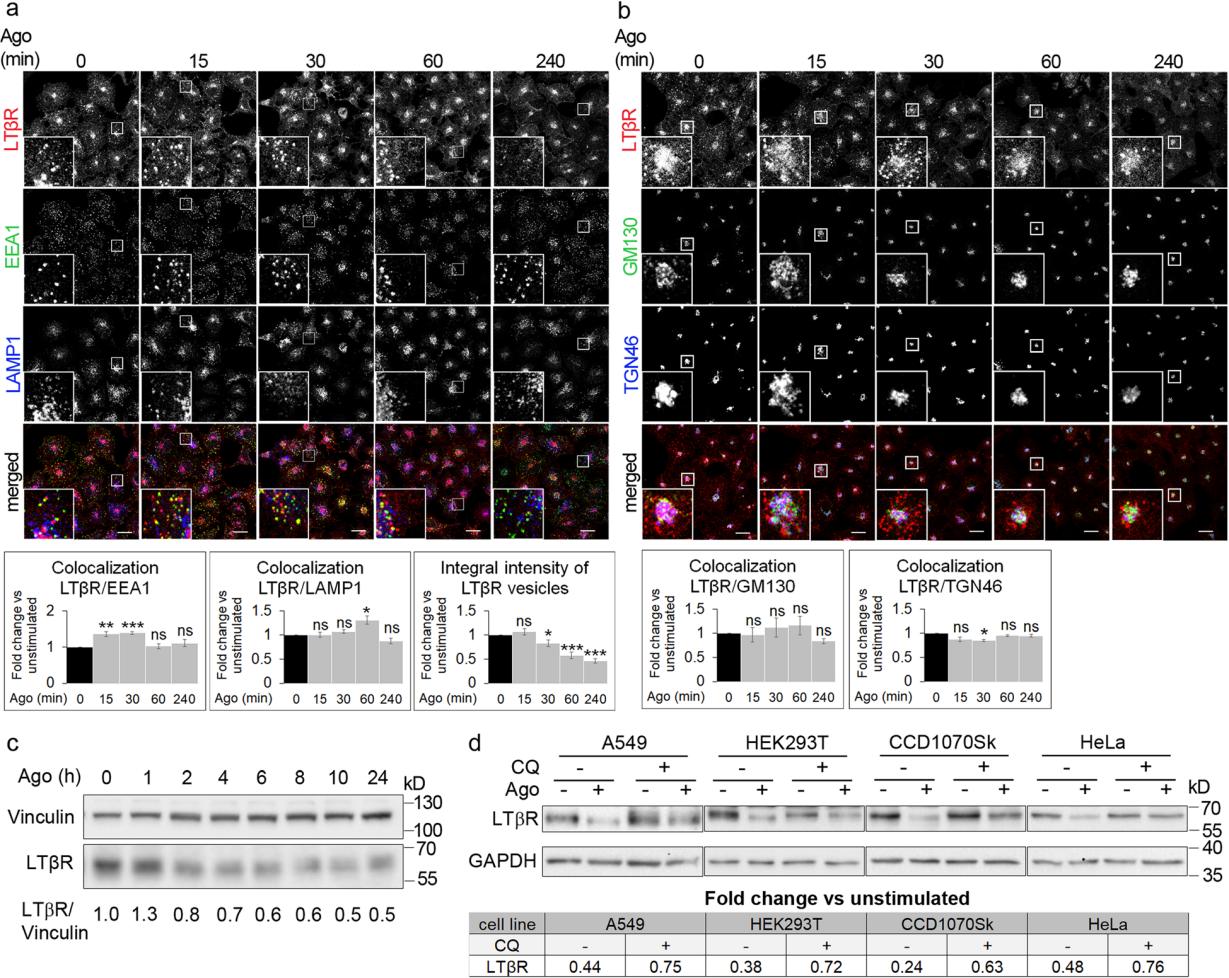
LTβR is internalized and trafficked towards degradation upon ligand binding. A549 cells were stimulated with Ago for the indicated time periods and immunostained for LTβR, EEA1 and LAMP1 (**a**) or trans- (TGN46) and cis-Golgi (GM130) (**b**). Insets show magnified views of boxed regions in the main images. Scale bars, 20 μm. Graphs represent the analysis of colocalization between LTβR and EEA1 or LAMP1, and integral intensity of LTβR (**a**) and colocalization between LTβR and GM130 or TGN46 (**b**). Data represent the means ± SEM, *n* ≥ 5 (**a**), *n* = 3 (**b**); ns - *P* > 0.05; *P ≤ 0.05; **P ≤ 0.01; ***P ≤ 0.001 by one sample *t* test. **c** Lysates of A549 cells stimulated with Ago for different time periods were analyzed by Western blotting with antibodies against LTβR and vinculin (used as a loading control). Representative blots are shown. Values below blots represent the averaged LTβR/vinculin ratio (*n* = 5) in cells stimulated with Ago for the indicated time periods. Values are normalized to unstimulated control (time 0) set as 1.**d** Lysates of A549, HEK293T, CCD1070Sk and HeLa cells pretreated or not for 20 h (A549, HEK293T, CCD1070Sk) or 16 h (HeLa) with lysosomal degradation inhibitor, chloroquine (CQ), stimulated or not with Ago for the next 4 h were analyzed by Western blotting with antibodies against LTβR and GAPDH (used as a loading control). Representative blots are shown. Table presents the fold change of LTβR abundance in stimulated vs unstimulated cells (means, *n* ≥ 3)

In our previous studies we noticed a substantial intracellular pool of LTβR which exhibits a complex distribution under basal (unstimulated) conditions in diverse cell lines (HeLa, HEK293 and A549) [36, 59, 60]. More specifically, we observed large perinuclear LTβR-positive structures, in addition to vesicular staining. To identify intracellular compartments occupied by LTβR in our model A549 cell line, we performed a systematic microscopic analysis of cells stained for LTβR and different markers of endosomes (early endosomes: EEA1, late endosomes/lysosomes: LAMP1, Fig. 1a) or the Golgi apparatus (cis-Golgi: GM130, trans-Golgi: TGN46, Fig. 1b). We investigated cells unstimulated (0 min) and stimulated with agonistic anti-LTβR antibody (Ago) for different time periods (15–240 min) to assess if and how the intracellular distribution of LTβR is affected by ligand binding. The quantitative analysis of microscopic images confirmed that under unstimulated conditions LTβR localized to early and late endosomes [59–61]. We found that the colocalization of LTβR with EEA1 peaked at 15–30 min of stimulation with Ago, whereas with LAMP1 at 60 min (Fig. 1a). Moreover, we noticed that the large perinuclear pool of the receptor colocalizing with Golgi markers was unaffected by administration of Ago (Fig. 1b). Integral intensity of LTβR vesicles, a parameter reflecting the amounts of the receptor present specifically in vesicular structures, decreased after 30 min of incubation with the ligand and subsequently dropped almost by half after 240 min (Fig. 1a). Western blot analysis of LTβR level revealed its progressive decrease starting from 2 h of stimulation (Fig. 1c). This, together with the colocalization of LTβR with the marker of late endosomes/lysosomes, suggested that ligand administration promoted lysosomal degradation of the receptor. Our hypothesis was evaluated in cells pretreated with chloroquine (CQ), a compound inhibiting the activity of lysosomal enzymes by preventing acidification of endosomal compartments. Following the treatment, A549, HEK293T, CCD1070Sk and HeLa cells were unable to reduce the amounts of LTβR upon stimulation with Ago as efficiently as control untreated cells (Fig. 1d). It was manifested with higher fold changes of LTβR level in stimulated vs unstimulated cells upon incubation with CQ than under normal conditions (Table in Fig. 1 d).

Our results show that LTβR, regardless of the stimulation status of the cell, is constitutively present on early and late endosomes as well as the Golgi apparatus. However, ligation of the receptor modulates its intracellular distribution and leads to its degradation in lysosomes.

### Ligand-bound LTβR is trafficked towards lysosomes

**Fig. 2:**
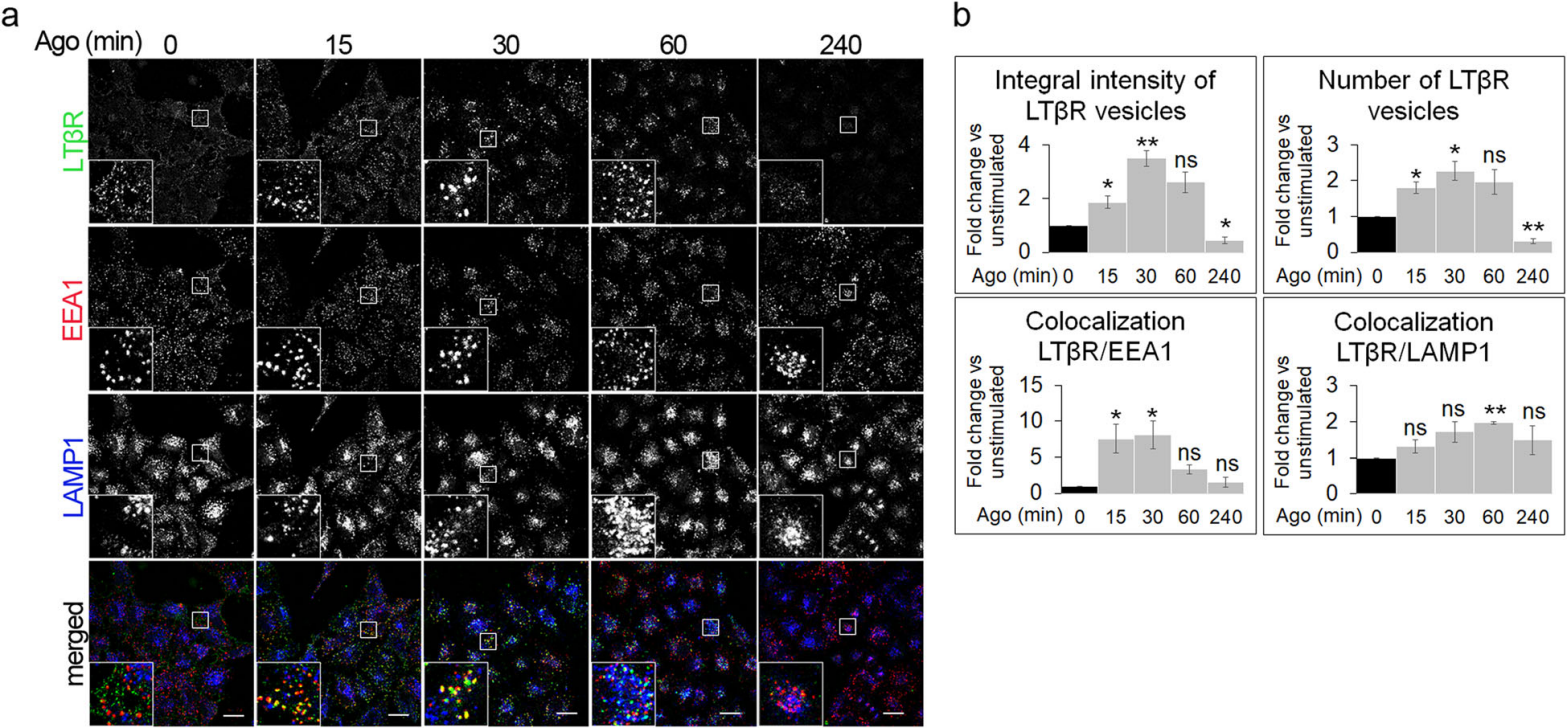
Ligand-bound LTβR localizes to endocytic compartments. **a** A549 cells were stimulated with Ago for the indicated time periods and immunostained for ligand-bound LTβR, EEA1 and LAMP1. Insets show magnified views of boxed regions in the main images. Scale bars, 20 μm. **b** Graphs represent the analysis of integral intensity and number of LTβR vesicles, and colocalization between LTβR and EEA1 or LAMP1. Data represent the means ± SEM, *n* = 4; ns - *P* > 0.05; *P ≤ 0.05; **P ≤ 0.01 by one sample *t* test

Since LTβR is constitutively present on membranes inside the cell, the effect of stimulation on its intracellular distribution and trafficking shown above could be underestimated. Thus, to eliminate this constitutive background we performed microscopic analyses to detect specifically a ligand-bound pool of the receptor. Cells, stimulated as described above, were stained for endocytic markers and the receptor associated with Ago (Fig. 2a), according to our previously optimized protocol [36]. Quantitative analysis of microscopic data revealed significant changes in the integral intensity and the number of LTβR-positive vesicles during stimulation, with the highest values at 30 min (Fig. 2b). We also confirmed that the localization of Ago-bound LTβR on early endosomes was the highest at 30 min of stimulation, whereas its localization on late endosomes/lysosomes peaked at 60 min (Fig. 2b). Since we did not observe any staining of intracellular ligand-bound receptor at 240 min (Fig. 2a), we concluded that it was degraded within lysosomes. Thus, the decrease in integral intensity of LTβR-positive vesicles showed in Fig. 1a and in receptor protein level in Fig. 1c and d, reflected the degradation of ligand-bound fraction of LTβR.

Taken together, our results show that upon ligand binding LTβR is internalized and trafficked via early endosomes to late endosomes/lysosomes for the degradation.

### LTβR internalization depends on clathrin and dynamin

We and others previously showed that both ligand-free and ligand-bound LTβR undergoes internalization [36, 59–61]. Recently, we demonstrated that depletion of cavin-1 (regulator of caveolae formation) only partially reduced internalization of ligand-bound receptor [36]. Thus, we concluded that another endocytic route/routes should be involved in LTβR trafficking. To identify them, we depleted cells of known regulators of different endocytic pathways using RNAi. Then, cells deprived of particular proteins were stimulated with Ago, stained for ligand-bound LTβR and EEA1 and analyzed by confocal microscopy. Having characterized the kinetics of LTβR trafficking upon ligand stimulation (Fig. 2b), we measured amounts of the internalized ligand-bound receptor (integral intensity) and the number of vesicles occupied by it upon 30 min of stimulation, when both parameters reached the maximal values (Fig. 2b).

**Fig. 3:**
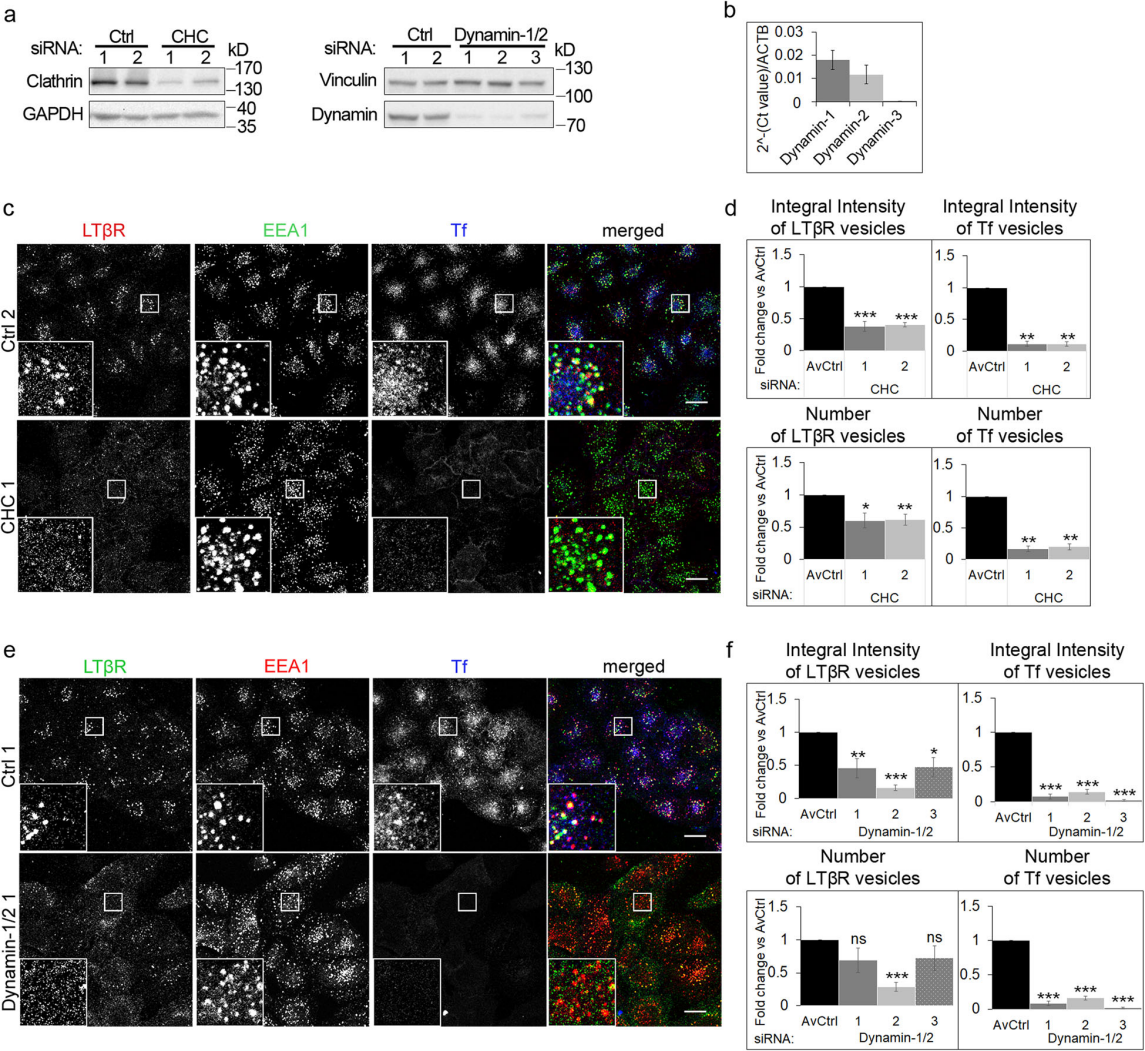
Ligand-bound LTβR is internalized through clathrin-mediated and dynamin-dependent endocytosis. **a** A549 cells were transfected with siRNAs targeting clathrin (CHC, two oligonucleotides denoted with consecutive numbers) and dynamin-1/2 (three combinations of oligonucleotides targeting dynamin-1 and dynamin-2, see Methods) along with non-targeting control (Ctrl) siRNAs (two oligonucleotides or two combinations of oligonucleotides, see Methods). The knock-down efficiency was analyzed by Western blotting with the relevant antibodies. Vinculin and GAPDH were used as loading controls. Representative blots are shown. **b** mRNA levels of transcripts encoding: dynamin-1, dynamin-2 and dynamin-3 isoforms were analyzed in A549 cells by qRT-PCR. Data represent the means ± SEM, *n* = 4. **c, e** A549 cells were depleted of clathrin (**c**) or dynamin-1/2 (**e**), incubated with Ago and fluorescent transferrin (Tf) for 30 min and immunostained with antibodies against ligand-bound LTβR and EEA1. Insets show magnified views of boxed regions in the main images. Scale bars, 20 μm. Images show representative examples of cells transfected with one (out of two or three) relevant targeting or non-targeting siRNA denoted with numbers described in the Methods section. **d, f** Graphs represent quantitative analysis of microscopic images from experiments exemplified in c and e with respect to integral intensity and number of LTβR- and Tf-positive vesicles. Data represent the means ± SEM, *n* ≥ 3; averaged non-targeting controls (AvCtrl) set as 1; ns - *P* > 0.05; *P ≤ 0.05; **P ≤ 0.01; ***P ≤ 0.001 by one sample *t* test

First, we investigated the contribution of CME to LTβR internalization. To this end, we knocked down expression of genes encoding clathrin heavy chain or dynamin-1 and dynamin-2 (Fig. 3a), as both dynamin isoforms were expressed at relatively high levels in A549 cells (Fig. 3b). Depletion of these proteins inhibited internalization of transferrin (Tf), a classical cargo of CME [69, 70], as evidenced by ~ 80% decrease of integral intensity and the number of Tf-positive vesicles, in comparison to control cells transfected with non-targeting siRNAs (Fig. 3c-f). This testified to efficient blocking of endocytic routes dependent on clathrin and dynamin. Under these conditions, the internalization of LTβR was reduced roughly by half, judging by the integral intensities and numbers of LTβR-positive vesicles (Fig. 3d, f). However, a degree of reduction varied between cells transfected with different combinations of siRNAs targeting dynamins (Fig. 3f).

To confirm the above results, we blocked CME with alternative methods, using chemical inhibitors: chlorpromazine (CPZ, CME inhibitor [71]) and dynasore (DYN, dynamin inhibitor [72]). We observed a profound inhibition of Tf and LTβR internalization in cells treated with these compounds (Additional file 2: Fig. S1a, b).

Yet another way to block CME was to deprive cells of the clathrin adaptor AP2M1 [38]. To this end, we employed a CRISPR/Cas9 genome editing technology, but despite clonal selection, we failed to obtain cells with the complete knock-out (Additional file 2: Fig. S1c). Nevertheless, even partial depletion of AP2M1 reduced both, Tf and LTβR internalization, roughly by half (Additional file 2: Fig. S1d). This finding clearly indicated that LTβR constitutes a cargo recognized by AP2M1, that confirmed a previous observation of Ganeff et al., who found the receptor co-immunoprecipitated with AP2M1 [61]. The interaction between these two proteins probably involves a dileucine motif (localized at the C-terminal part of the receptor [61]), which was postulated to be important for cargo recognition by the AP2 complex [73].

Taken together, our results indicate the involvement of clathrin- and dynamin-dependent routes in LTβR internalization. Since efficient blocking of these routes did not completely prevent the internalization of LTβR, it is plausible that other parallel endocytic pathways are employed by the receptor.

### LTβR is internalized through CIE pathways

**Fig. 4:**
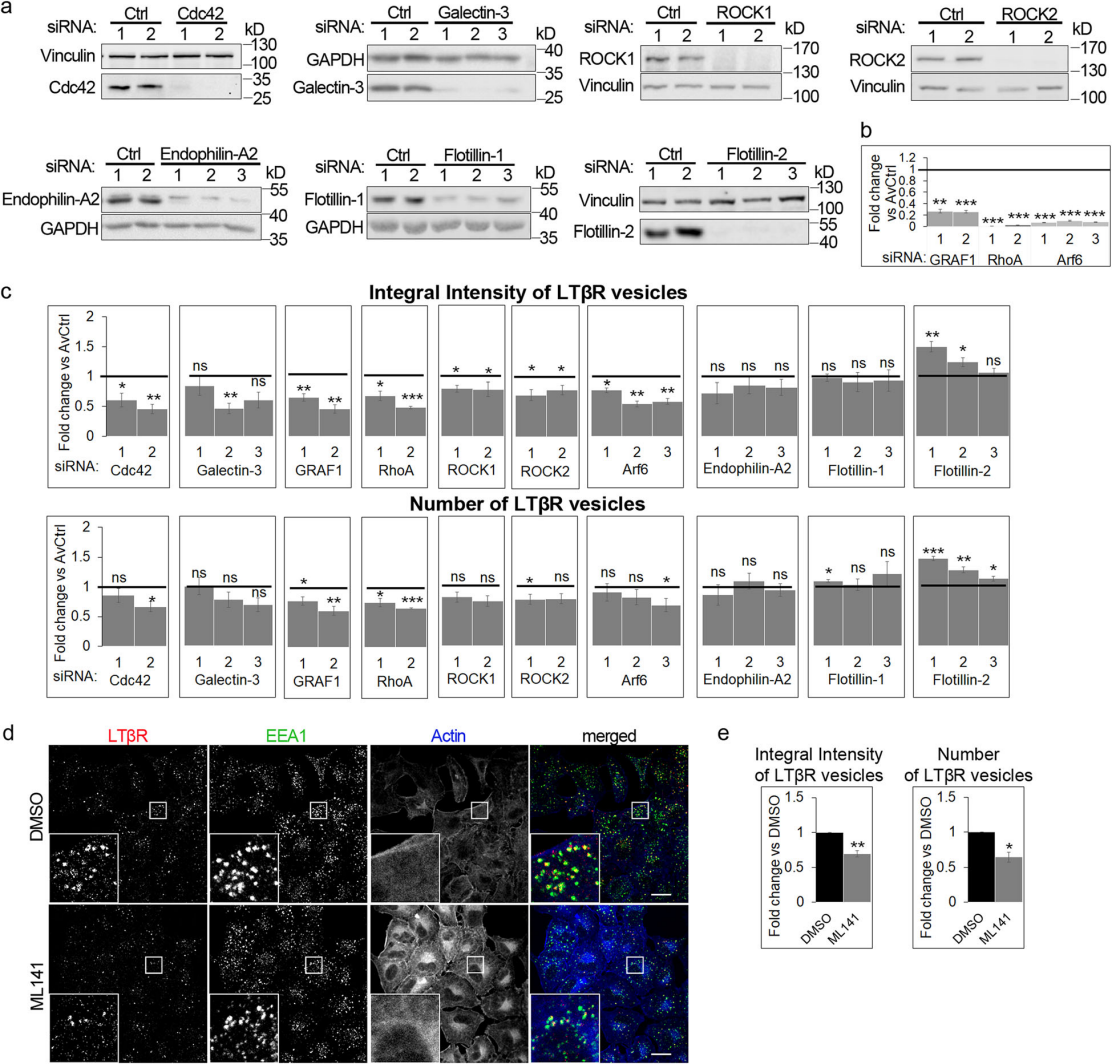
Ligand-bound LTβR is internalized through CIE routes. **a** A549 cells were transfected with siRNAs (two or three oligonucleotides per gene, denoted with consecutive numbers) against Cdc42, galectin-3, ROCK1, ROCK2, endophilin-A2, flotillin-1, flotillin-2, along with non-targeting control (Ctrl) siRNAs (two oligonucleotides denoted with consecutive numbers) and analyzed by Western blot for knock-down efficiency. Vinculin and GAPDH were used as loading controls. Representative blots are shown. **b** A549 cells were depleted of GRAF1, RhoA or Arf6 using siRNAs (two or three oligonucleotides per gene, denoted with consecutive numbers), along with non-targeting (Ctrl) siRNAs (two oligonucleotides) and analyzed by qRT-PCR with respect to the knock-down efficiency of GRAF1, RhoA or Arf6, respectively. Values are presented as a fold change vs averaged non-targeting controls (AvCtrl), set as 1 (line). Data represent the means ± SEM, n = 3; **P ≤ 0.01; ***P ≤ 0.001 by one sample *t* test. **c** A549 cells depleted of the indicated endocytic regulators with siRNAs (at least two siRNAs per gene, denoted with consecutive numbers) and stimulated for 30 min with Ago were immunostained for the ligand-bound LTβR and imaged using confocal microscopy. Graphs represent the analysis of the resulting images with respect to integral intensity (top) and number (bottom) of vesicles marked with ligand-bound LTβR. Values are presented as fold change vs AvCtrl set as 1 (line). Data represent the means ± SEM, n ≥ 3; ns - *P* > 0.05; *P ≤ 0.05; **P ≤ 0.01; ***P ≤ 0.001 by one sample *t* test. **d** A549 cells were treated with DMSO or ML141, stimulated with Ago for 30 min and immunostained for the ligand-bound LTβR and EEA1. Actin was stained with phalloidin. Insets show magnified views of boxed regions in the main images. Scale bars, 20 μm. **e** Graphs represent quantitative analysis of microscopic images from experiments exemplified in d with respect to integral intensity and number of LTβR-positive vesicles. Values are presented as fold change vs DMSO set as 1. Data represent the means ± SEM, n = 4; *P ≤ 0.05; **P ≤ 0.01 by one sample *t* test

To identify alternative or additional endocytic route/routes followed by LTβR, we used an analogical strategy to the one described above. We measured internalization of ligand-bound LTβR upon impairment of diverse routes of CIE [46], achieved by depletion of their key regulators (Fig. 4a, b). We aimed to impair: CLIC/GEEC (regulated by Cdc42, galectin-3 and GRAF1), RhoA-dependent route (regulated by RhoA, ROCK1, ROCK2), Arf6-associated endocytosis, FEME (marked by endophilin-A2), as well as those dependent on the presence of flotillin-1 and flotillin-2. We observed a decrease of the integral intensity and the number of LTβR-positive vesicles in cells deprived of Cdc42, GRAF1, RhoA, ROCK1, ROCK2 and Arf6, as well as, for single siRNAs, of galectin-3 (Fig. 4c). Endophilin-A2 and flotillin-1 did not seem to be crucial for LTβR internalization since their knock-down (Fig. 4a) did not alter either the integral intensity or the number of LTβR vesicles (Fig. 4c). In turn, depletion of flotillin-2 increased both analyzed parameters.

To further confirm that LTβR is internalized via CLIC/GEEC, we used Cdc42 inhibitor, ML141, which inhibits GTP binding to this protein [74, 75]. In A549 cells, we examined the status of actin filaments (as their polymerization dynamics depends on Cdc42 [76]), to corroborate the efficacy of Cdc42 inhibition by ML141 (Fig. 4d). Cells treated with the compound exhibited disturbed actin filaments and reduced LTβR internalization (Fig. 4d, e). Moreover, the effects of Cdc42 inhibition (Fig. 4e) and depletion (Fig. 4c) on the integral intensity and the number of LTβR-positive vesicles were comparable.

Cumulatively, our results indicate that LTβR internalization occurs through diverse endocytic pathways, which include: CME, Cdc42-regulated CLIC/GEEC, RhoA-dependent and Arf6-associated routes.

### The NF-κB pathway is preferentially activated by LTβR ligands in A549 cells

**Fig. 5:**
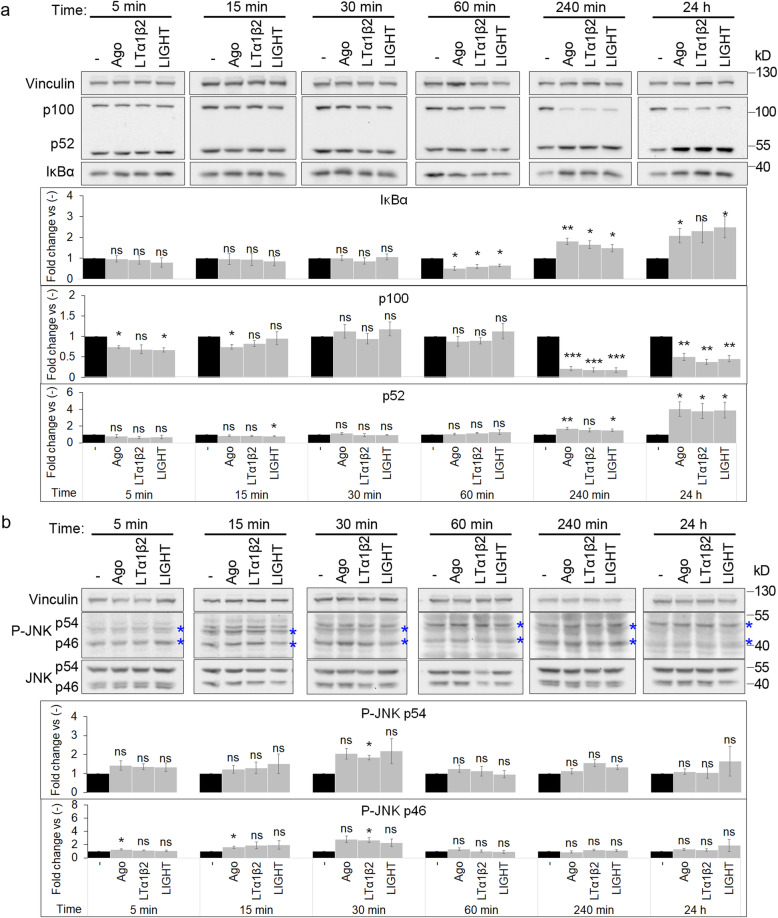
Stimulation of LTβR with ligands preferentially activates NF-κB signaling. Lysates of A549 cells stimulated with: Ago, LTα1β2 and LIGHT for different time periods were analyzed by Western blotting with antibodies against the indicated proteins to assess activity of the NF-κB pathway (**a**) and JNK-dependent AP-1 pathway; P-JNK - phospho-JNK; analyzed bands marked with asterisks (**b**). Graphs show densitometric analysis of abundance of the indicated proteins, normalized to vinculin, used as a loading control. Values are presented as a fold change vs unstimulated control (−), set as 1. Data represent the means ± SEM, n ≥ 3; ns - *P* > 0.05; *P ≤ 0.05; **P ≤ 0.01; ***P ≤ 0.001 by one sample *t* test. Representative blots are shown

Although LTβR was shown to trigger different signaling pathways [10–14], the kinetics of their activation by LTβR ligands was not studied systematically. To address this issue, we analyzed the status of selected pathways in A549 cells stimulated for various time periods with Ago, LTα1β2 or LIGHT. We observed that despite minor differences between three ligands, in general they acted in a similar manner (Fig. 5a, b).

With respect to the kinetics of the NF-κB pathway activation, the degradation of IκBα inhibitory protein (a hallmark of the canonical branch) was observed at 60 min after ligand administration. In later time points we observed an increase in the level of this protein (Fig. 5a), which confirmed the pathway activation, as IκBα-encoding *NFKBIA* gene is a known NF-κB target [77]. The processing of p100 to transcriptionally potent p52 (a hallmark of the non-canonical NF-κB branch) could be observed at 240 min of stimulation and continued for the next several hours (Fig. 5a). These data confirmed observations from our previous study [36].

We also investigated the kinetics of activation of JNK-dependent AP-1 pathway. We observed only a slight increase of JNK phosphorylation at Thr183/Tyr185 (that reflects its activation) at 30 min of stimulation with ligands (Fig. 5b).

Finally, we checked the activation of Akt and ERK1/2-dependent signaling and the status of STAT1 and STAT3 transcription factors. Akt and ERK1/2 activities, assessed based on the levels of their phosphorylation (Thr202/Tr204 and Ser473, respectively), were not affected under the examined conditions (Additional file 2: Fig. S2a). We also did not observe any significant changes in the activation of STAT1 or STAT3 measured by phosphorylation at Tyr701 and Tyr705, respectively (Additional file 2: Fig. S2b).

Together, our observations indicate that ligand binding by LTβR preferentially triggers the NF-κB and, to a small extent, JNK-dependent AP-1 pathways. Thus, we decided to focus on NF-κB signaling, which seems to be the major pathway activated by LTβR in A549 cells.

In parallel to biochemical analyses, we characterized the kinetics of expression of genes encoding cytokines (*CCL20, TNF, IL6, CXCL8, CCL5, CCL2*), cell adhesion molecules (*ICAM1, VCAM1*), growth factor (*CSF2*) and NF-κB pathway regulators (*NFKBIA, NFKB2, RELB*) that were previously shown to be up-regulated in response to LTβR stimulation in A549 cells [36]. We found that the expression of some genes (*CCL20, TNF, IL6, CXCL8, CCL2*) increased early, at 1–4 h after ligand administration, while others (*CCL5, ICAM1, VCAM1, CSF2, NFKBIA, NFKB2, RELB*) were up-regulated upon longer treatment (Additional file 2: Fig. S3). Moreover, the expression patterns in cells stimulated with different LTβR ligands were similar (Additional file 2: Fig. S3), which confirmed the results of biochemical analyses mentioned before (Fig. 5a). Based on the obtained data we selected two time periods of stimulation with Ago (2 and 24 h) for further studies.

### Blocking clathrin- and dynamin-dependent LTβR internalization increases activation of the canonical NF-κB pathway

We previously demonstrated that certain defects in intracellular trafficking of LTβR can lead to strong ligand-independent activation of NF-κB signaling [59]. Furthermore, in our recent study we showed that cholesterol removal from the PM restricted internalization of LTβR and led to strong activation of the canonical branch of the NF-κB pathway in a ligand-dependent manner [36]. To gain better insights into the link between intracellular trafficking and signaling, we investigated if internalization of LTβR via the identified endocytic routes influenced its signaling potential. For this purpose, we depleted cells of Cdc42, clathrin, dynamin-1/2, or used relevant inhibitors: ML141, chlorpromazine or dynasore, since under these conditions internalization of ligand-bound LTβR was affected the most (Fig. 3, Fig. 4 and Additional file 2: Fig. S1). Following siRNA-mediated knock-down or chemical inhibition of the selected proteins we evaluated the status of the NF-κB pathway.

**Fig. 6:**
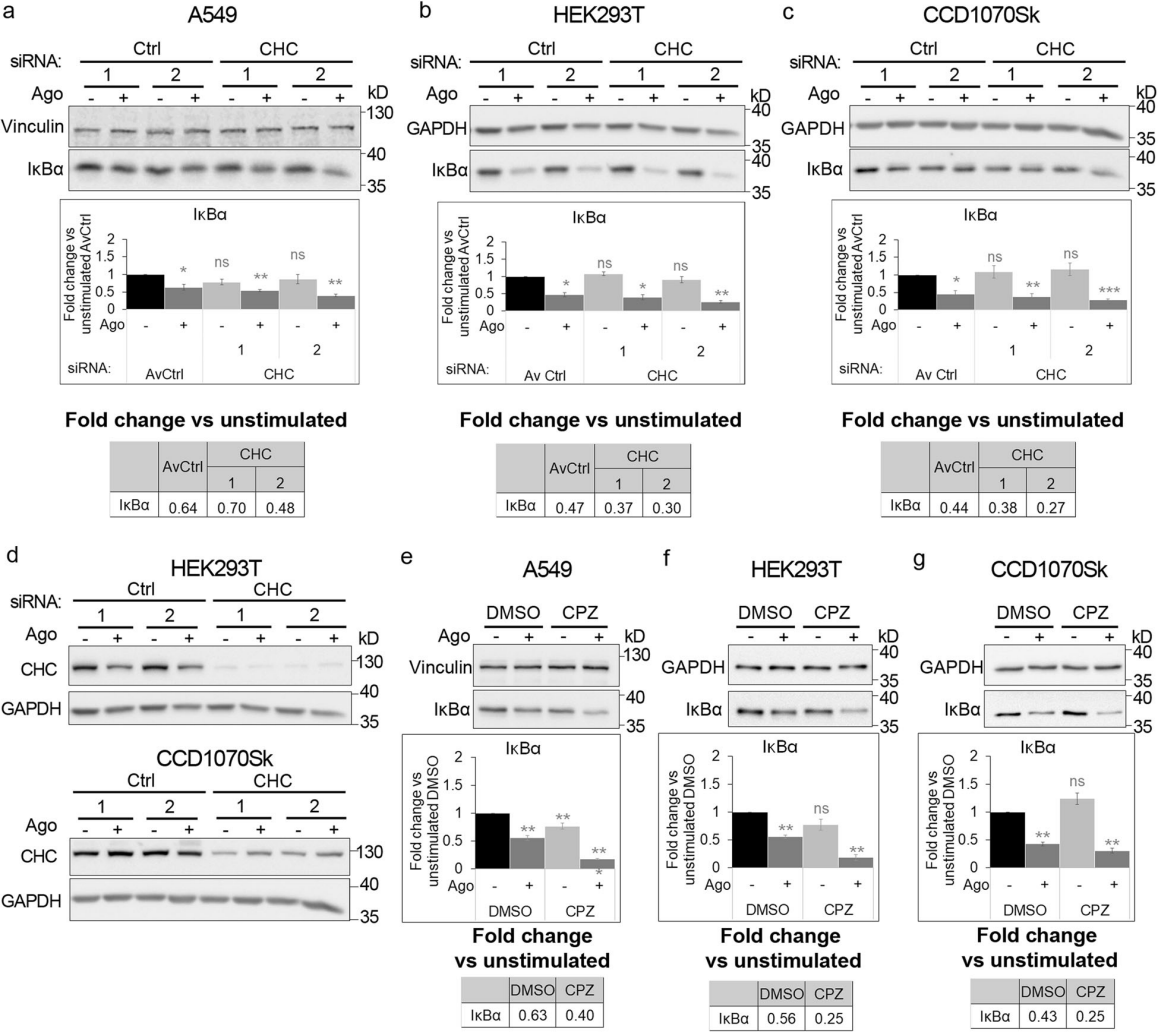
Blocking clathrin-dependent endocytosis enhances activation of canonical NF-κB signaling by LTβR. A549 (**a**), HEK293T (**b, d**) and CCD1070Sk (**c, d**) cells were transfected with siRNAs targeting clathrin (CHC) (two oligonucleotides) along with control, non-targeting siRNAs (two oligonucleotides) or treated with chlorpromazine (CPZ, **e-g**) along with DMSO, and stimulated or not with Ago for 1 h. Lysates of cells were analyzed by Western blotting with antibodies against the indicated proteins. Graphs show densitometric analysis of abundance of IκBα, normalized to loading controls (GAPDH or vinculin). Values are presented as a fold change vs unstimulated non-targeting controls – averaged non-targeting controls (AvCtrl) or DMSO, set as 1. Data represent the means ± SEM, n = 3 (**a**, **b**, **f**, **g**), n = 4 (**c**, **e**); ns - *P* > 0.05; *P ≤ 0.05; **P ≤ 0.01; ***P ≤ 0.001 by one sample *t* test. Tables present the fold change of IκBα abundance in stimulated vs unstimulated cells (means, n ≥ 3). **d** HEK293T and CCD1070Sk cells were analyzed with respect to the efficiency of clathrin knock-down. Representative blots are shown. The blots of GAPDH shown in panels **b** and **c** are also shown in panel **d**

**Fig. 7:**
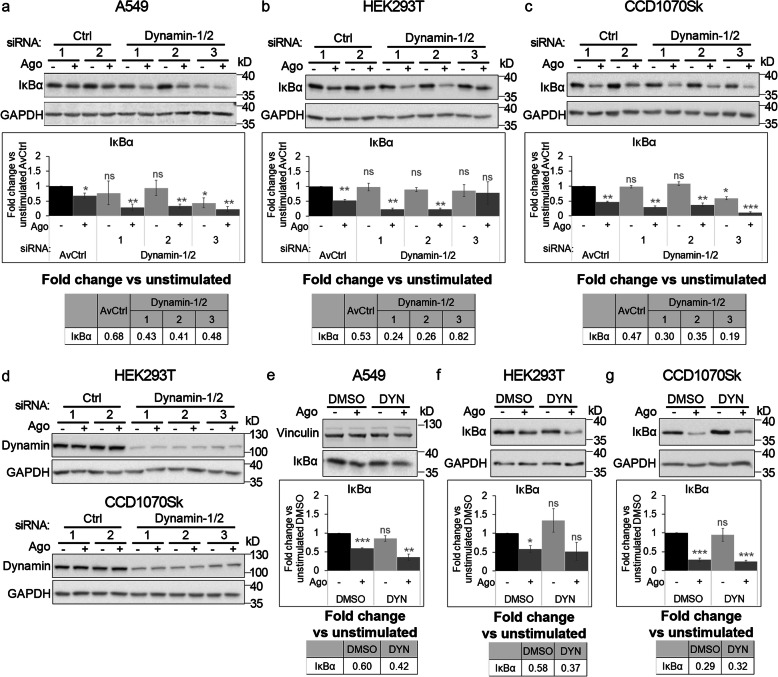
Blocking dynamin-dependent endocytosis enhances activation of canonical NF-κB signaling by LTβR. A549 (**a**), HEK293T (**b, d**) and CCD1070Sk (**c, d**) cells were transfected with siRNAs targeting dynamin-1/2 (three combinations of oligonucleotides targeting dynamin-1 and dynamin-2, see Methods) along with non-targeting control (Ctrl) siRNAs (two combinations of oligonucleotides, see Methods) or treated with dynasore (DYN, **e-g**) along with DMSO, and stimulated or not with Ago for 1 h. Lysates of cells were analyzed by Western blotting with antibodies against the indicated proteins. Graphs show densitometric analysis of abundance of IκBα, normalized to loading controls (GAPDH or vinculin). Values are presented as a fold change vs unstimulated non-targeting controls – averaged non-targeting controls (AvCtrl) or DMSO, set as 1. Data represent the means ± SEM, n = 3 (**b**, **c**, **f**), n = 4 (**a**, **e**, **g**); ns - *P* > 0.05; *P ≤ 0.05; **P ≤ 0.01; ***P ≤ 0.001 by one sample *t* test. Tables present the fold change of IκBα abundance in stimulated vs unstimulated cells (means, n ≥ 3). **d** HEK293T and CCD1070Sk cells were analyzed with respect to the efficiency of dynamin knock-down. Representative blots are shown

We found that depletion of Cdc42 did not affect the level of IκBα (the canonical NF-κB branch) in unstimulated cells. In turn, degradation of IκBα in response to LTβR stimulation (represented by fold change vs unstimulated conditions, see Table in Additional file 2: Fig. S4a) was either reduced or potentiated, depending on siRNA used for silencing. Thus, to verify Cdc42 effect on activation of LTβR-triggered canonical NF-κB signaling, we treated cells with ML141. Administration of the inhibitor had no effect on the levels of IκBα under basal conditions or upon Ago stimulation (Additional file 2: Fig. S4b), denoting that the activation of the canonical NF-κB pathway did not depend on Cdc42. In turn, clathrin depletion potentiated degradation of IκBα in response to Ago administration in A549 cells (transfected with one of two targeting siRNAs, Fig. 6a). It was confirmed in non-cancer HEK293T and non-transformed fibroblast CCD1070Sk cells (transfected with either of targeting siRNAs, Fig. 6b and c), despite differences in clathrin depletion efficiency between these cell lines (Fig. 6d). This, together with the fact that proteolysis of IκBα was enhanced in Ago stimulated cells upon treatment with chlorpromazine (Fig. 6e-g), suggested that CME may regulate the canonical branch of the NF-κB pathway. Similar results were obtained in dynamin-deficient cells, in which stimulation with Ago led to stronger degradation of the pathway inhibitor in comparison to control cells (Fig. 7a-c) in all tested cell lines, regardless of the efficiency of dynamin knock-down (Fig. 3a and Fig. 7d). Also the treatment with dynasore led to stronger degradation of IκBα in A549 and HEK293T cells (Fig. 7e, f), although it did not seem to affect CCD1070Sk cells (Fig. 7g). The basal levels of IκBα remained unchanged in cells depleted of dynamin-1/2 (Fig. 7a-c, except one combination of siRNAs in A549 and CCD1070Sk cells) or treated with dynasore (Fig. 7e-g). All these data indicate that canonical NF-κB signaling driven by LTβR activation depends on clathrin- and dynamin-mediated internalization, but not Cdc42-regulated endocytosis.

### Blocking clathrin- and dynamin-dependent LTβR internalization reduces activation of the non-canonical NF-κB pathway

**Fig. 8:**
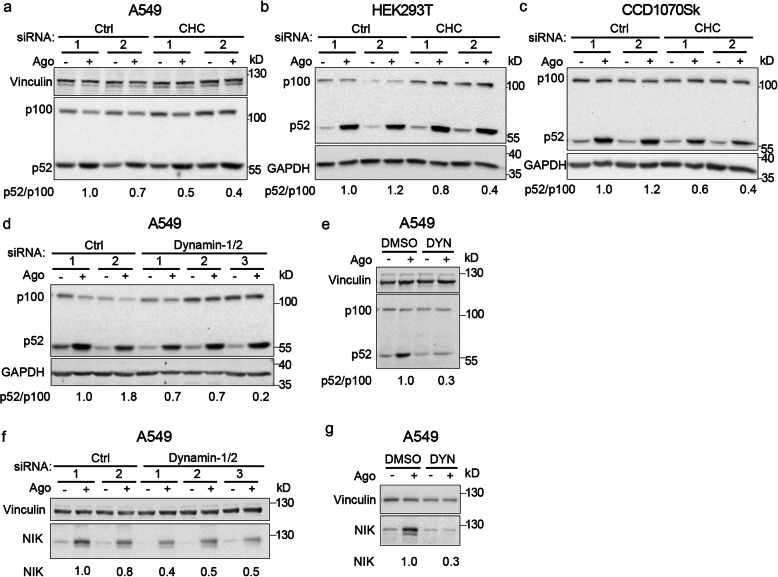
Clathrin and dynamin deficiency reduces activation of the non-canonical NF-κB pathway. **a-c** A549, HEK293T and CCD1070Sk cells were transfected with siRNAs targeting clathrin (CHC) (two oligonucleotides) along with control, non-targeting siRNAs (two oligonucleotides) and stimulated or not with Ago for 1 h. **d, f** A549 cells were transfected with siRNAs targeting dynamin-1/2 (three combinations of oligonucleotides targeting dynamin-1 and dynamin-2, see Methods) along with non-targeting control (Ctrl) siRNAs (two combinations of oligonucleotides, see Methods) and stimulated or not with Ago for 1 h. **e, g** A549 cells were treated with dynasore (DYN) or DMSO and stimulated or not with Ago for 1 h. Lysates of cells were analyzed by Western blotting with antibodies against the indicated proteins. Representative blots are shown. Values presented below blots represent the averaged p52/p100/loading control (**a**-**e**) or NIK/loading control ratio (f-g) from at least three experiments (normalized to the selected control, set as 1) in cells stimulated with Ago

In parallel, we evaluated how impairment of the abovementioned endocytic routes affected the activation of non-canonical NF-κB signaling. To this end, we analyzed the status of crucial components of the pathway in cells incubated with Ago for 24 h. We focused on the processing of p100 to p52, presented as the normalized p52/p100 ratio, and the levels of NIK. We noticed that the results from cells depleted of Cdc42 and those treated with Cdc42 inhibitor (ML141) were not coherent. In Cdc42-depleted cells the p52/p100 ratio was higher than in controls (2.3 and 1.6 vs 1.0 and 1.1), whereas it was lower in ML141-treated cells (0.5 vs 1.0) (Additional file 2: Fig. S5a, b). These data did not allow us to determine a link between Cdc42-regulated endocytosis and non-canonical NF-κB signaling triggered by LTβR. In turn, clathrin depletion reduced p100 processing (lower p52/p100 ratio) in response to LTβR stimulation in all tested cell lines (Fig. 8a-c). Similarly, dynamin-1/2 depletion or inhibition with dynasore reduced p100 processing in response to LTβR stimulation in A549 cells (Fig. 8d and e). This confirmed previous findings of Ganeff et al. about the role of dynamin in promoting non-canonical NF-κB signaling [61]. Since the reduced p52/p100 ratio in dynamin-depleted A549 cells might partially result from increased level of p100 observed upon dynamin knock-down (Fig. 8d), we assessed the status of an upstream regulator of the non-canonical NF-κB branch, NIK protein. We found that silencing of dynamin-1/2-encoding genes reduced Ago-dependent accumulation of NIK (Fig. 8f), which in turn was abolished by dynasore treatment (Fig. 8g).

Our data provided evidence that clathrin-dependent endocytosis is important for non-canonical NF-κB signaling in diverse cellular models, including carcinoma A549, non-cancer, transformed HEK293T, and non-transformed fibroblast CCD1070Sk cells. We also confirmed the previous finding about importance of dynamin-dependent internalization for the non-canonical NF-κB pathway [61].

### LTβR target gene expression is changed upon clathrin and dynamin depletion

**Fig. 9:**
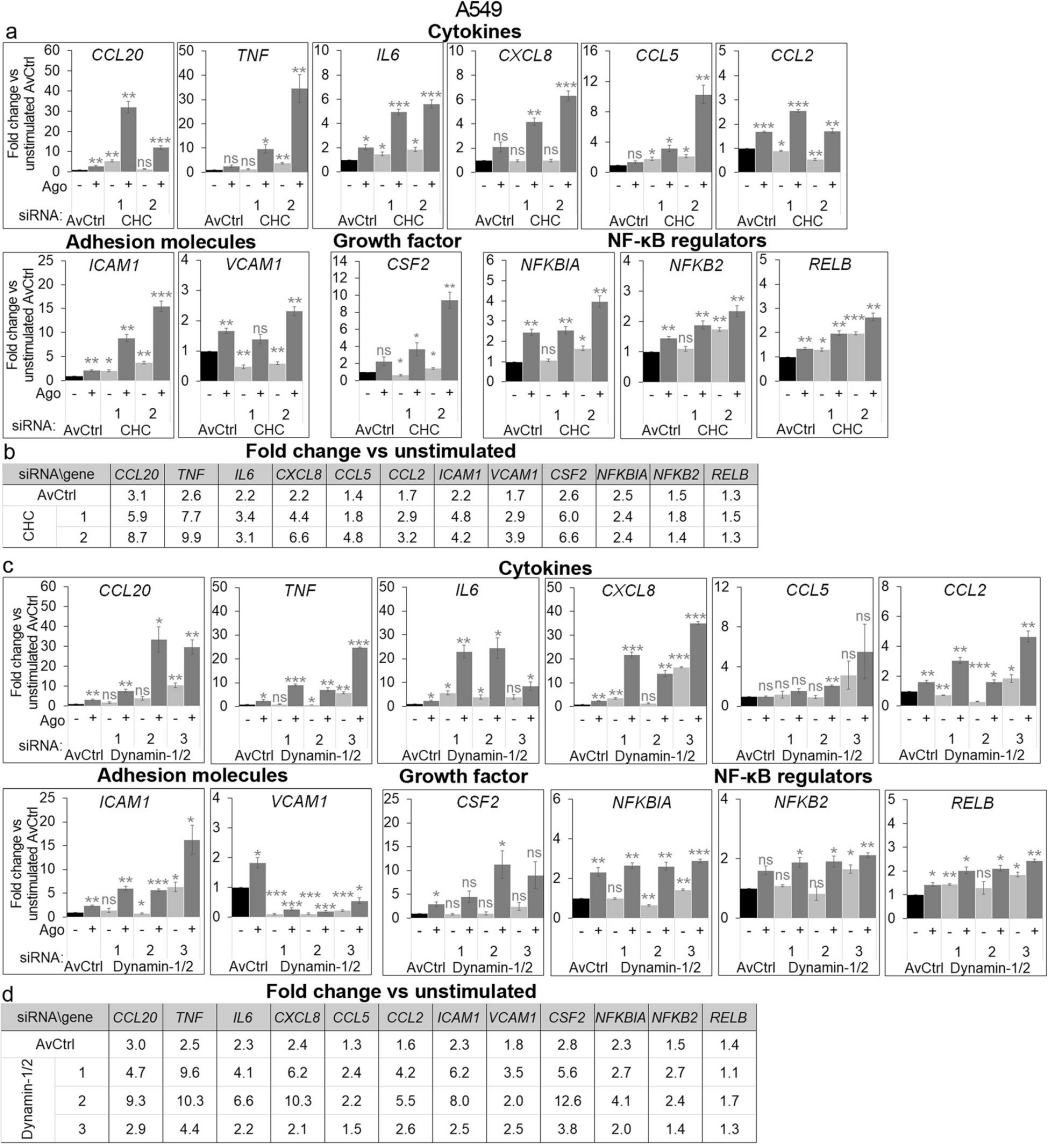
Depletion of clathrin and dynamin enhances expression of LTβR target genes in A549 cells. mRNA levels of NF-κB target genes were analyzed by qRT-PCR in A549 cells transfected with siRNAs targeting clathrin (CHC, two oligonucleotides denoted with consecutive numbers) (**a**, **b**), dynamin-1/2 (three combinations of oligonucleotides targeting dynamin-1 and dynamin-2, see Methods) (**c**, **d**) and with relevant non-targeting siRNAs and stimulated with Ago for 2 h. Values are presented as a fold change vs unstimulated averaged non-targeting controls (AvCtrl), set as 1. Data represent the means ± SEM, n ≥ 3; ns - *P* > 0.05; *P ≤ 0.05; **P ≤ 0.01; ***P ≤ 0.001 by one sample *t* test. Tables present the fold change of expression of the indicated genes in stimulated vs unstimulated cells transfected with different combinations of siRNAs, targeting clathrin (**b**) and dynamin-1/2 (**d**), and non-targeting controls

To further corroborate our observations, we examined if enhanced LTβR-dependent activation of the canonical NF-κB pathway upon clathrin and dynamin-1/2 depletion corresponded to changes in the transcription of LTβR target genes. We measured the levels of previously selected transcripts in A549 and HEK293T cells depleted of clathrin or dynamin-1/2 and stimulated or not with Ago for 2 h (Fig. 9 and Additional file 2: Fig. S6). We found that expression of some genes in these cells was changed even under unstimulated conditions in comparison to controls (Fig. 9a, c and Additional file 2: Fig. S6a, c). Moreover, the effect of clathrin and dynamin-1/2 knock-down on the transcription of selected genes was cell-type specific, as expected. Thus, to assess the effect of clathrin and dynamin-1/2 depletion specifically on the responsiveness of cells to LTβR stimulation, we calculated the fold change of expression in stimulated vs unstimulated conditions in controls and cells deprived of clathrin or dynamin-1/2 (Fig. 9b, d and Additional file 2: Fig. S6b, d). In agreement with our data presented in Additional file 2: Fig. S3, 2 h stimulation of control A549 cells (transfected with non-targeting siRNAs) with Ago led to a significant increase in the expression of early genes (e.g. *CCL20, TNF, IL6, CXCL8, CCL2).* This increase varied from 1.7 for *CCL2* up to 3.1 fold for *CCL20* in control A549 cells (designated as AvCtrl) from experiments with clathrin depletion (Fig. 9b). Similar increase was observed upon dynamin-1/2 knock-down (1.6 for *CCL2*, and 3.0 for *CCL20*, Fig. 9d), although here we applied a combination of non-targeting siRNAs. The results obtained for HEK293T cells confirmed data from A549 cell line with minor differences regarding the set of genes affected (Additional file 2: Fig. S6b, d). Comparison of expression fold changes (stimulated vs unstimulated) between control and clathrin- or dynamin-depleted cells revealed that following the knock-down, cells were more responsive to the receptor stimulation. Although there were differences between clathrin- and dynamin-deficient cells, and between cells transfected with different siRNAs, the data clearly showed increase in expression of the majority of the examined genes that was further enhanced upon 2 h of stimulation. The most pronounced effects of endocytosis impairment in A549 cells were observed for early expressed genes, e.g. *TNF* and *CXCL8*. We noticed 2.5 fold increase of *TNF* transcript levels in response to stimulation in control A549 cells, whereas in dynamin-depleted cells this increase varied from 4.4 up to 10.3 fold depending on siRNA combination (Fig. 9d). Although displaying some cell type-specific changes in comparison to A549, HEK293T cells were also sensitized to stimulation with Ago upon silencing of clathrin- and dynamin-1/2-encoding genes (Additional file 2: Fig. S6b, d).

We also examined the impact of clathrin and dynamin-1/2 deprivation on transcription pattern upon prolonged stimulation with Ago in A549 cells. We found that 24 h after Ago administration the expression of *VCAM1* and *CSF2* genes was preferentially upregulated in control cells (AvCtrl, Additional file 2: Fig. S7). In clathrin- and dynamin-1/2-depleted cells expression of only selected genes (e.g. *CCL20, CCL5, ICAM1*) was enhanced in comparison to controls upon 24 h stimulation with Ago (Additional file 2: Fig. S7b, d).

Taken together, our results indicate that the expression of LTβR target genes depends on its endocytosis. The inhibition of LTβR internalization potentiates the responsiveness of cells to stimulation of the receptor with the ligand, that is manifested at the level of effector proteins and gene expression.

## Discussion

### LTβR follows distinct endocytic routes

It is commonly accepted that endocytosis can influence cellular localization, protein levels, as well as signaling potential of receptors. While many of them were shown to use multiple internalization routes [21, 38], the purpose of such variation is vague. Some receptors use different endocytic routes for activation of specific signaling, e.g. TNFR1-dependent NF-κB pathway activation was shown to depend on clathrin, but not on caveolae [78]. The latter were shown to regulate the activation of RhoA [79] and the PI3K/Akt pathway [80] triggered by the receptor stimulation. In this study we identified the routes of ligand-bound LTβR internalization and the consequences of their blocking for signaling triggered by the receptor. So far only a few studies of others and our own pointed to the role of dynamin- and cavin-1-dependent endocytosis for LTβR function [36, 61]. Our data indicate that LTβR enters a cell mostly via pathways dependent on the activity of dynamins. However, their depletion did not fully abolish LTβR internalization, while it almost completely blocked uptake of canonical cargo, Tf. This fact urged us to search for alternative or additional endocytic pathways employed by the receptor. By performing systematic microscopic analysis of LTβR internalization we demonstrated that LTβR follows multiple endocytic routes to enter a cell upon ligand binding. They include CME, as well as several pathways of CIE: dynamin-independent CLIC/GEEC (since Cdc42 and GRAF1 depletion partially reduced LTβR internalization), and dynamin-dependent routes involving RhoA, ROCK1, ROCK2, and Arf6. In addition, and to our surprise, we found that silencing of flotillin-2 caused increased internalization of ligand-bound LTβR. Based on a similar observation for ErbB2, it was suggested that flotillins rather stabilize this receptor at the PM [81], instead of promoting its internalization. We cannot exclude that the same mechanism operates in case of LTβR, however we did not examine this scenario further within this study.

We postulate that cells employ distinct endocytic routes to ensure efficient internalization of LTβR. It is likely that keeping the receptor amounts at the PM at relatively low levels minimizes unnecessary stimulation of the receptor at the cell surface by subthreshold stimuli. It was proposed that the impairment of endocytic machinery found in glioma cells might lead to prolonged RTK-dependent signaling [82]. In case of LTβR, endocytosis via multiple routes could constitute a mechanism of protection against over-activation of LTβR-evoked pro-inflammatory responses, that can be detrimental if not controlled [83]. Moreover, we showed that upon binding of the ligands LTβR is sent towards degradation in lysosomes, which reduces its cellular levels. Previously, it was demonstrated that both local accumulation on the limiting membrane of endosomes [59] and overexpression [84] of LTβR activate pro-inflammatory signaling even in the absence of a ligand, suggesting that a precise mechanism controlling endocytosis of LTβR is crucial.

### Signaling triggered by LTβR depends on its internalization

In our recently published study we showed that the reduction of LTβR internalization, by acute depletion of the PM cholesterol, enhances LTβR-triggered pro-inflammatory canonical NF-κB signaling [36]. Here we show that ligand-dependent activation of the canonical NF-κB branch is enhanced upon impairment of dynamin-dependent endocytosis (in cells depleted of dynamin-1 and dynamin-2, or treated with dynasore) and upon blocking of CME (by clathrin depletion or treatment with chlorpromazine). This new finding supported our previous observation that there is a correlation between receptor uptake and its signaling potential [36]. However, it is in contrast to the conclusion made by Ganeff et al., who claimed that only the non-canonical branch of NF-κB signaling depended on dynamin-regulated endocytosis [61]. They did not observe hyper-activation of the canonical NF-κB pathway (judged by enhanced degradation of IκBα inhibitor) in their model HeLa cells, either upon dynamin depletion/inhibition, or upon clathrin deprivation. Discrepancy between the present study and the one published by Ganeff et al. could be explained by the use of different cellular models and time periods of receptor stimulation selected for analyses.

Enhanced expression of target genes supported our conclusion about the stronger activation of the canonical NF-κB route in dynamin- and clathrin-deprived cells. It seems that increase in the levels of most examined transcripts in response to LTβR stimulation (fold change stimulated vs unstimulated cells) was higher in cells with impaired endocytosis in comparison to control cells (Fig. 9 & Additional file 2: Fig. S6 and S7). We observed that the calculated fold changes correlated to some extent with the degree of the reduction of LTβR internalization. A549 cells transfected with the combination of dynamin-targeting siRNAs which limited LTβR uptake the most effectively (dynamin-1/2 2; Fig. 3f) exhibited the highest responsiveness to the receptor stimulation (Fig. 9). The effect of clathrin- and dynamin-mediated endocytosis impairment on ligand-dependent gene expression was observed mostly for those transcripts, which under conditions of normal endocytosis increased within first 4 h of the stimulation (Additional file 2: Fig. S3 & Fig. 9). It may suggest that transcriptional response reflects the activation of immediately triggered signaling pathways, operating via NF-κB or AP-1 transcription factors. Moreover, our data show enhanced expression of selected targets upon silencing of clathrin or dynamins under ligand-free conditions. This confirms previously reported findings concerning the stimulatory effect of clathrin depletion or dominant negative dynamin-2 mutant on gene transcription [85, 86]. The sensitivity of clathrin- and dynamin-depleted A549 cells to LTβR ligand manifests itself more at the transcriptional level (Fig. 9 & Additional file 2: Fig. S7) than at the stage of NF-κB pathway activation (measured by degradation of pathway inhibitor IκBα, Fig. 6a & Fig. 7a). As we showed that both NF-κB and JNK-dependent AP-1 pathways [87] are triggered in A549 cells upon ligand stimulation (Fig. 5), it is possible that the observed difference is a result of an additive effect of activation of distinct signaling pathways.

With respect to the activation of the non-canonical branch of the NF-κB pathway, A549 cells partially recapitulated the results obtained in HeLa cell line by Ganeff et al. [61]. A549 cells deprived of dynamins or treated with dynasore were unable to process p100 to p52 and accumulate NIK as efficiently as it was observed in controls in response to ligand administration (Fig. 8d-g). A more pronounced effect on NIK levels exerted by the treatment with dynasore might be explained by efficient blocking of dynamin activity by dynasore that cannot be achieved by siRNA-mediated knock-down. It is possible that the remaining dynamin molecules in siRNA-transfected cells can still sustain residual activity, that enables some accumulation of NIK. However, it could be insufficient to promote full processing of p100 to p52. This would indicate that under normal conditions dynamin is involved in regulation of the non-canonical NF-κB pathway at different stages: upstream and downstream from NIK. In contrast to the previously published data [61], our study revealed that clathrin-depleted cells exhibited impaired processing of p100 to p52 in response to stimulation with Ago (Fig. 8a-c). These results indicate that CME is also important for the activation of the non-canonical NF-κB pathway.

Altogether, our study demonstrates that perturbation in clathrin- and dynamin-dependent endocytosis increases cell responsiveness to LTβR stimulation, that leads to the stronger activation of the canonical branch of NF-κB signaling. We propose that under normal conditions a pro-inflammatory response to LTβR ligation is restricted by the receptor internalization via diverse endocytic routes, that prevents prolonged residence of the receptor at the cell surface. Impairment of clathrin- and dynamin-dependent endocytosis increases the availability of the receptor for ligand binding at the PM. This in turn enhances LTβR-triggered canonical NF-κB and JNK-dependent AP-1 signaling together with transcription of early genes, as it was shown for EGFR-dependent Akt signaling [88]. Alternatively, clathrin and dynamin depletions might activate these pathways in a ligand-independent manner, making cells ‘primed’ for ligand-dependent signaling. It was recently shown that macrophages with elevated basal level of NF-κB were more sensitive and faster responding to infection [89]. With respect to the non-canonical NF-κB pathway, our data support the idea proposed by Ganeff et al. [61]. According to it, dynamin-dependent endocytosis of LTβR is crucial for activation of non-canonical branch by the internalized receptor present at the surface of endosomes. We postulate, that under perturbation of endocytosis (either clathrin- or dynamin-dependent) reduced levels of endosomal LTβR are insufficient to fully activate non-canonical branch of the NF-κB pathway. Thus, in cells deprived of clathrin or dynamins, enhancement of transcriptional response to prolonged LTβR stimulation (observed for selected targets, Additional file 2: Fig. S7) most probably reflects hyper-activation of the canonical branch. Moreover, we cannot exclude that upon impairment of endocytosis LTβR affects other signaling pathways (in addition to NF-κB or AP-1), not examined within this study, that in consequence could regulate expression of specific target genes.

Our data hint at existence of a complex endocytosis-related regulatory machinery of LTβR-dependent signaling that still is not fully understood. Recognizing the network of pathways affected by LTβR endocytosis could broaden our understanding of the receptor biology and direct us towards possible applications of the current data, e.g. in receptor-targeting therapies.

## Supplementary information

**Additional file 1 Table 1.** List of primary antibodies. Abbreviations: WB-Western blotting, IF-Immunofluorescence. **Table 2.** List of primers for qRT-PCR. **Table 3.** List of sgRNA sequences

**Additional file 2 Figure S1** Ligand-bound LTβR is internalized through CME**.** A549 cells treated with DMSO or chlorpromazine (CPZ, **a**), dynasore (DYN, **b**) or depleted of AP2M1 through CRISPR/Cas9 genome editing (two non-targeting, NT and two AP2M1 targeting sgRNAs denoted with consecutive numbers) (**d**) were incubated with Ago and transferrin (Tf) for 30 min and immunostained for the ligand-bound LTβR and EEA1. Insets show magnified views of boxed regions in the main images. Scale bars, 20 μm. Graphs represent quantitative analysis of microscopic images from experiments exemplified in a, b, and d with respect to integral intensity and number of LTβR- and Tf-positive vesicles. Data represent the means ± SEM, *n* = 5 (a), *n* = 6 (b), *n* = 3 (d). Values are presented as fold change vs DMSO (a, b) or averaged non-targeting controls (AvNT) (d) set as 1; ns - *P* > 0.05; *P ≤ 0.05; **P ≤ 0.01; ***P ≤ 0.001 by one sample *t* test. **c** Knock-down efficiency of AP2M1 in A549 cells transfected with sgRNAs (two sequences targeting AP2M1 and two non-targeting, NT) and non-transfected, was analyzed by Western blot. Representative blots and images are shown. **Figure S2** Stimulation with LTβR ligands does not activate Akt, ERK1/2, STAT1 or STAT3**.** Lysates of A549 cells stimulated with: Ago, LTα1β2 and LIGHT for different time periods were analyzed by Western blotting with antibodies against the indicated proteins to assess activity of Akt and ERK1/2 (**a**), and STAT1 and STAT3 (**b**). P-Akt - phospho-Akt; P-ERK1/2 - phospho-ERK1/2; P-STAT1 - phospho-STAT1; P-STAT3 - phospho-STAT3. The blots of vinculin (loading control) in a are also used in lower part of panel b. The blots of vinculin in the upper part of panel b are also shown in Fig. 5b. Graphs show densitometric analysis of the abundance of the indicated proteins, normalized to loading control. Values are presented as a fold change vs unstimulated control (−), set as 1. Data represent the means ± SEM, *n* ≥ 3; ns - *P* > 0.05; *P ≤ 0.05; **P ≤ 0.01 by one sample *t* test. Representative blots are shown. **Figure S3** Stimulation with LTβR ligands leads to expression of NF-κB target genes. mRNA levels of NF-κB target genes were analyzed by qRT-PCR in A549 cells stimulated with: Ago, LTα1β2 and LIGHT for different time periods. Values are presented as a fold change vs unstimulated cells. Data represent the means ± SEM, *n* = 3. **Figure S4** Cdc42 deficiency does not affect the activation of canonical NF-κB signaling by LTβR**.** A549 cells were: transfected with siRNAs targeting Cdc42 (two oligonucleotides) (**a**) or treated with ML141 (**b**), along with the relevant controls, non-targeting siRNAs (two oligonucleotides, Ctrl) (a) or DMSO (b), and stimulated or not with Ago for 1 h. Lysates of cells were analyzed by Western blotting with antibodies against the indicated proteins. Representative blots are shown. Graphs show densitometric analysis of abundance of IκBα, normalized to loading control (GAPDH). Values are presented as a fold change vs unstimulated non-targeting controls – averaged non-targeting controls (AvCtrl) or DMSO, set as 1. Data represent the means ± SEM, *n* = 4 (a), n = 3 (b); ns - *P* > 0.05; *P ≤ 0.05; **P ≤ 0.01 by one sample *t* test. Tables present the fold change of IκBα abundance in stimulated vs unstimulated cells (means, n ≥ 3). **Figure S5** Cdc42 deficiency does not affect the activation of non-canonical NF-κB signaling by LTβR. A549 cells were: transfected with siRNAs targeting Cdc42 (two oligonucleotides) (**a**) or treated with ML141 (**b**), along with the relevant controls, non-targeting siRNAs (two oligonucleotides, Ctrl) (a) or DMSO (b), and stimulated or not with Ago for 24 h. Lysates of cells were analyzed by Western blotting with antibodies against the indicated proteins. Representative blots are shown. Values presented below blots represent the averaged p52/p100/loading control ratio from at least three experiments (normalized to GAPDH, set as 1) in cells stimulated with Ago. **Figure S6** Depletion of clathrin and dynamin enhances expression of LTβR target genes in HEK293T cells. mRNA levels of NF-κB target genes were analyzed by qRT-PCR in HEK293T cells transfected with siRNAs targeting clathrin (CHC, two oligonucleotides denoted with consecutive numbers) (**a**, **b**), dynamin-1/2 (three combinations of oligonucleotides targeting dynamin-1 and dynamin-2, see Methods) (**c**, **d**) and with relevant non-targeting siRNAs and stimulated with Ago for 2 h. Values are presented as a fold change vs unstimulated averaged non-targeting controls (AvCtrl), set as 1. Data represent the means ± SEM, n = 3; ns - *P* > 0.05; *P ≤ 0.05; **P ≤ 0.01; ***P ≤ 0.001 by one sample *t* test. Tables present the fold change of expression of the indicated genes in stimulated vs unstimulated cells transfected with different combinations of siRNAs, targeting clathrin (b) and dynamin-1/2 (d), and non-targeting controls. **Figure S7** Depletion of clathrin and dynamin enhances expression of the selected LTβR target genes in A549 cell upon prolonged stimulation. mRNA levels of NF-κB target genes were analyzed by qRT-PCR in A549 cells transfected with siRNAs targeting clathrin (CHC) (two oligonucleotides) (**a**, **b**) or dynamin-1/2 (three combinations of oligonucleotides targeting dynamin-1 and dynamin-2, see Methods) (**c**, **d**) and stimulated with Ago for 24 h. Values are presented as a fold change vs unstimulated averaged non-targeting controls (AvCtrl), set as 1. Data represent the means ± SEM, n = 3; ns - *P* > 0.05; *P ≤ 0.05; **P ≤ 0.01; ***P ≤ 0.001 by one sample *t* test. Tables present the fold change of the indicated gene expression in stimulated vs unstimulated cells transfected with siRNAs, targeting clathrin (b) and dynamin-1/2 (d) and non-targeting controls.

## Data Availability

The datasets used and/or analyzed during the current study are available on reasonable request.

## Abbreviations

Ago: Agonistic anti-LTβR antibody
AP-1: Activator protein 1
AP2: Adaptor protein 2
CIE: Clathrin-independent endocytosis
CHC: Clathrin heavy chain
CLIC/GEEC: Clathrin-Independent carriers/glycosylphosphotidylinositol-anchored protein (GPI-AP) enriched compartments endocytosis
CME: Clathrin-mediated endocytosis
CPZ: Chlorpromazine hydrochloride
DYN: Dynasore
CQ: Chloroquine
EEA1: Early endosome antigen 1
ERK1/2: Extracellular signal–regulated kinase 1 and extracellular signal–regulated kinase 2
FEME: Fast endophilin mediated endocytosis
GM130: Golgi matrix protein 130 kDa
GPCR: G protein-coupled receptor
JNK: c-Jun N-terminal kinase
LAMP1: Lysosomal associated membrane protein 1
LIGHT: Homologous to lymphotoxin, exhibits inducible expression and competes with HSV glycoprotein D for binding to herpesvirus entry mediator, a receptor expressed on T lymphocytes
LTα1β2: Lymphotoxin α1β2
LTβR: Lymphotoxin β receptor
MAPK: Mitogen-activated protein kinase
NF-κB: Nuclear factor kappa-light-chain-enhancer of activated B cells
NIK: NF-κB-inducing kinase
PM: Plasma membrane
RTK: Receptor tyrosine kinase
STAT: Signal transducer and activator of transcription
Tf: Transferrin
TGN46: Trans-Golgi network protein 2
TNFRSF: Tumor necrosis factor receptor superfamily
TRAF: TNF receptor associated factor
