# Supplementary material for: Clathrin- and dynamin-dependent endocytosis limits canonical NF-κB signaling triggered by lymphotoxin β receptor
Source: Cell Commun Signal. 2020 Nov 4;18:176. doi: 10.1186/s12964-020-00664-0 (PMC7640449; doi:10.1186/s12964-020-00664-0)
Supplement: Supplementary file 2 — Additional file 1 Table 1. List of primary antibodies. Abbreviations: WB-Western blotting, IF-Immunofluorescence. Table 2. List of primers for qRT-PCR. Table 3. List of sgRNA sequences [file 12964_2020_664_MOESM2_ESM.docx]

**Additional file 1**

**Table 1.** List of primary antibodies. Abbreviations: WB-Western blotting, IF-Immunofluorescence

| Antigen | Supplier | Cat. no. | Source | Application |
| --- | --- | --- | --- | --- |
| Akt | Cell Signaling | 2920 | mouse | WB 1:1000 |
| AP2M1 | BD Biosciences | 611350 | mouse | WB 1:1000 |
| Cdc42 | Santa Cruz Biotechnology | sc-87 | rabbit | WB 1:200 |
| Clathrin heavy chain | BD Biosciences | 610499 | mouse | WB 1:5000 |
| Dynamin | BD Biosciences | 610245 | mouse | WB 1:1000 |
| EEA1 | BD Biosciences | 610457 | mouse | IF 1:1000 |
| EEA1 | Enzo Life Sciences | ALX-210-239 | rabbit | IF 1:400 |
| Endophilin-A2 | Abcam | ab97336 | rabbit | WB 1:500 |
| Flotillin-1 | Santa Cruz Biotechnology | sc-74566 | mouse | WB 1:1000 |
| Flotillin-2 | Santa Cruz Biotechnology | sc-28320 | mouse | WB 1:1000 |
| Galectin-3 | Santa Cruz Biotechnology | sc-20157 | rabbit | WB 1:1000 |
| GAPDH | Santa Cruz Biotechnology | sc-25778 | rabbit | WB 1:2000 |
| GM130 | BD Biosciences | 610822 | mouse | IF 1:200 |
| IκBα | Cell Signaling | 4814S | mouse | WB 1:1000 |
| LAMP1 | Sigma-Aldrich | L-1418 | rabbit | IF 1:200 |
| LTβR | Santa Cruz Biotechnology | sc-8375 | goat | WB 1:500 |
| LTβR | Thermo Fisher Scientific | PA5-47028 | goat | WB 1:500 |
| LTβR | R&D Systems | AF629 | goat | IF 1:100  Stimulation 1:1000  of 0.2 mg/ml stock |
| NIK | Cell Signaling | 4994S | rabbit | WB 1:500 |
| p100/p52 | Cell Signaling | 4882S | rabbit | WB 1:1000 |
| p44/42 MAPK (ERK1/2) | Cell Signaling | 9107 | mouse | WB 1:1000 |
| P-Akt (Ser473) | Cell Signaling | 9271 | rabbit | WB 1:1000 |
| P-p44/42 MAPK (ERK1/2) (Thr202/Tr204) | Cell Signaling | 9101 | rabbit | WB 1:1000 |
| P-SAPK/JNK (Thr183/Tyr185) | Cell Signaling | 9255 | mouse | WB 1:1000 |
| P-STAT1 (Tyr701) | Cell Signaling | 7649 | rabbit | WB 1:1000 |
| P-STAT3 (Tyr705) | Cell Signaling | 9131 | rabbit | WB 1:1000 |
| ROCK1 | Santa Cruz Biotechnology | sc-5560 | rabbit | WB 1:1000 |
| ROCK2 | Santa Cruz Biotechnology | sc-5561 | rabbit | WB 1:1000 |
| SAPK/JNK | Cell Signaling | 9252 | rabbit | WB 1:1000 |
| STAT1 | Cell Signaling | 9172 | rabbit | WB 1:1000 |
| STAT3 | Cell Signaling | 9139 | mouse | WB 1:1000 |
| TGN46 | Sigma-Aldrich | T7576 | rabbit | IF 1:200 |
| Vinculin | Sigma-Aldrich | V9131 | mouse | WB 1:5000 |

**Table 2.** List of primers for qRT-PCR

| **Gene** | **Forward primer** | **Reverse primer** |
| --- | --- | --- |
| *ACTB* | CAGGTCATCACCATTGGCAAT | TCTTTGCGGATGTCCACGT |
| *ARF6* | ATGGGGAAGGTGCTATCCAAAATC | GCAGTCCACTACGAAGATGAGACC |
| *ARHGAP26 (GRAF1)* | TAAGAATGCTTCCAGGACCACTC | GCTGTAACATCTGCCGATTTTTC |
| *B2M* | GGAGGCTATCCAGCGTACTC | GAAACCCAGACACATAGCAATTC |
| *CCL2* | gaagaatcaccagcagcaag | cttggccacaatggtcttga |
| *CCL20* | CTGGCTGCTTTGATGTCAGT | CGTGTGAAGCCCACAATAAA |
| *CCL5* | ACCAGTGGCAAGTGCTCCAAC | CTCCCAAGCTAGGACAAGAGCAAG |
| *CSF2* | CATGATGGCCAGCCACTACAA | ACTGGCTCCCAGCAGTCAAAG |
| *CXCL8* | GCTCTCTTGGCAGCCTTCCTGA | TTTCCTTGGGGTCCAGACAGAGC |
| *DNM1* | gctgactgctgagaatctgtcc | gtgtctcacaggctagctccag |
| *DNM2* | Catgatcctgcagttcatcagc | cttctcaacgggagcaacttgt |
| *DNM3* | ACTCCAGCCAACACTGATCTTGC | CCCCTGCGAAGAGGCAACAGT |
| *ICAM1* | GGAGCCCGCTGAGGTCACGA | AGTCGCTGGCAGGACAAAGGT |
| *IL6* | gggctcttcggcaaatgtag | gaaggaatgcccattaacaacaa |
| *NFKB2* | GCTGGAGGAGGCGGGCGTCTAA | GGGCTGGCTCCTTGGGTTCCA |
| *NFKBIA* | CGCCCAAGCACCCGGATACA | AGGGCAGCTCGTCCTCTGTGA |
| *RELB* | GGAAAGACTGCACCGACGGCA | TTCAGGGACCCAGCGTTGTAGGG |
| *RHOA* | CAGAAAAGTGGACCCCAGAA | GCAGCTGCTCTCGTAGCCATTTC |
| *TNF* | GTGATCGGCCCCCAGAGGGA | TGAGGGTTTGCTACAACATGGGC |
| *VCAM1* | CCGGATTGCTGCTCAGATTGGA | AGCGTGGAATTGGTCCCCTCA |

**Table 3.** List of sgRNA sequences

| **sgRNA** | **Sequence (5’->3’)** |
| --- | --- |
| AP2M1 1 | CACCGGAGAGGGTATCAAGTATCGT |
| AP2M1 2 | CACCGACTTGCTGAGTCGCACACAC |
| NT1 | CACCGCTGAAAAAGGAAGGAGTTGA |
| NT2 | CACCGAAGATGAAAGGAAAGGCGTT |
