# Supplementary material for: Clathrin- and dynamin-dependent endocytosis limits canonical NF-κB signaling triggered by lymphotoxin β receptor
Source: Cell Commun Signal. 2020 Nov 4;18:176. doi: 10.1186/s12964-020-00664-0 (PMC7640449; doi:10.1186/s12964-020-00664-0)
Supplement: Supplementary file 3 — Additional file 2 Figure S1 Ligand-bound LTβR is internalized through CME. A549 cells treated with DMSO or chlorpromazine (CPZ, a), dynasore (DYN, b) or depleted of AP2M1 through CRISPR/Cas9 genome editing (two non-targeting, NT and two AP2M1 targeting sgRNAs denoted with consecutive numbers) (d) were incubated with Ago and transferrin (Tf) for 30 min and immunostained for the ligand-bound LTβR and EEA1. Insets show magnified views of boxed regions in the main images. Scale bars, 20 μm. Graphs represent quantitative analysis of microscopic images from experiments exemplified in a, b, and d with respect to integral intensity and number of LTβR- and Tf-positive vesicles. Data represent the means ± SEM, n = 5 (a), n = 6 (b), n = 3 (d). Values are presented as fold change vs DMSO (a, b) or averaged non-targeting controls (AvNT) (d) set as 1; ns - P > 0.05; *P ≤ 0.05; **P ≤ 0.01; ***P ≤ 0.001 by one sample t test. c Knock-down efficiency of AP2M1 in A549 cells transfected with sgRNAs (two sequences targeting AP2M1 and two non-targeting, NT) and non-transfected, was analyzed by Western blot. Representative blots and images are shown. Figure S2 Stimulation with LTβR ligands does not activate Akt, ERK1/2, STAT1 or STAT3. Lysates of A549 cells stimulated with: Ago, LTα1β2 and LIGHT for different time periods were analyzed by Western blotting with antibodies against the indicated proteins to assess activity of Akt and ERK1/2 (a), and STAT1 and STAT3 (b). P-Akt - phospho-Akt; P-ERK1/2 - phospho-ERK1/2; P-STAT1 - phospho-STAT1; P-STAT3 - phospho-STAT3. The blots of vinculin (loading control) in a are also used in lower part of panel b. The blots of vinculin in the upper part of panel b are also shown in Fig. 5b. Graphs show densitometric analysis of the abundance of the indicated proteins, normalized to loading control. Values are presented as a fold change vs unstimulated control (−), set as 1. Data represent the means ± SEM, n ≥ 3; ns - P > 0.05; *P ≤ 0.05; **P ≤ 0.01 by one [file 12964_2020_664_MOESM3_ESM.docx]

**Additional file 2**

**
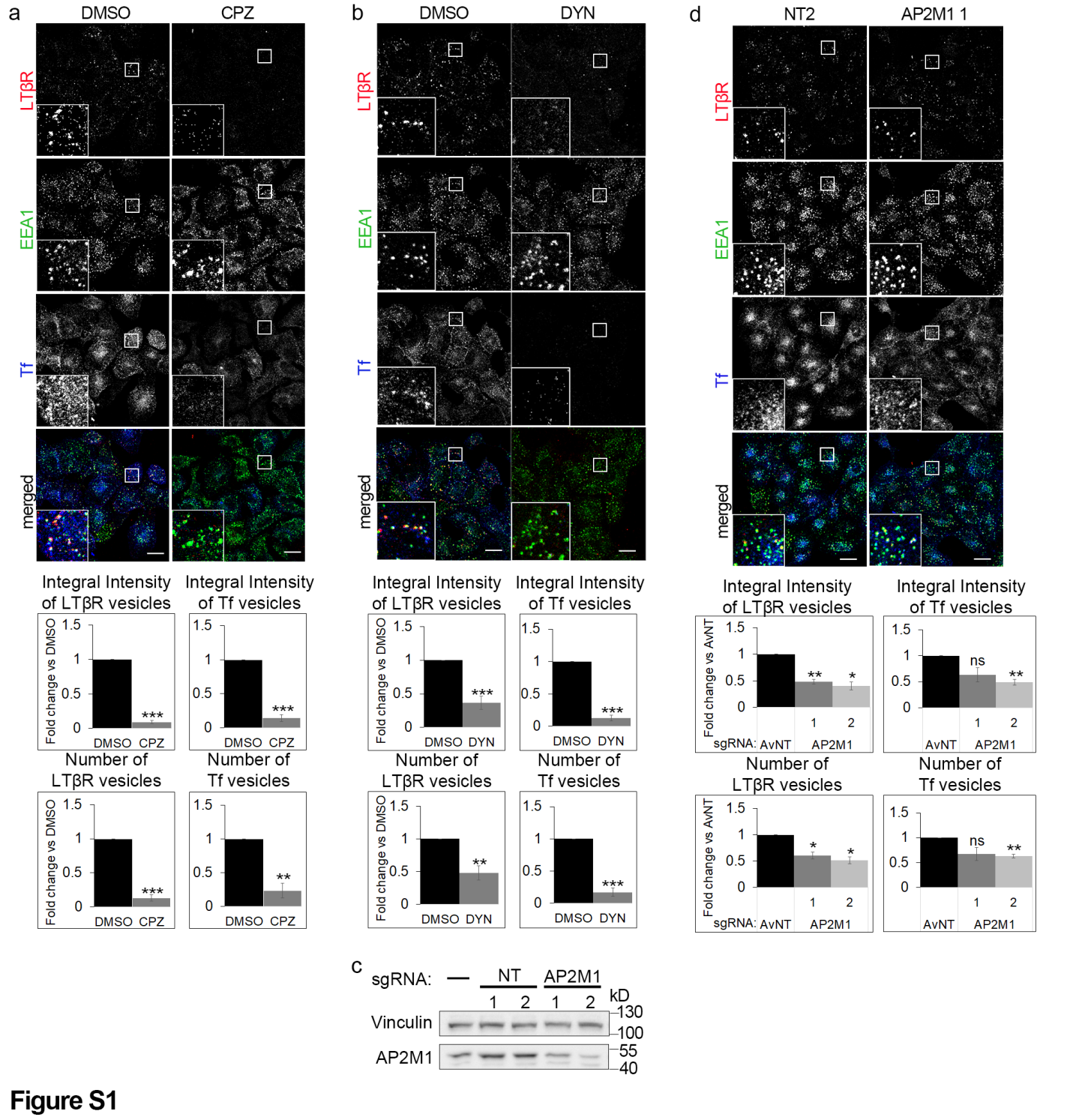
**

**Figure S1 Ligand-bound LTβR is internalized through CME.**

A549 cells treated with DMSO or chlorpromazine (CPZ, **a**), dynasore (DYN, **b**) or depleted of AP2M1 through CRISPR/Cas9 genome editing (two non-targeting, NT and two AP2M1 targeting sgRNAs denoted with consecutive numbers) (**d**) were incubated with Ago and transferrin (Tf) for 30 min and immunostained for the ligand-bound LTβR and EEA1. Insets show magnified views of boxed regions in the main images. Scale bars, 20 µm. Graphs represent quantitative analysis of microscopic images from experiments exemplified in a, b, and d with respect to integral intensity and number of LTβR- and Tf-positive vesicles. Data represent the means ± SEM, n=5 (a), n=6 (b), n=3 (d). Values are presented as fold change vs DMSO (a, b) or averaged non-targeting controls (AvNT) (d) set as 1; ns - P>0.05; *P≤0.05; **P≤0.01; ***P≤0.001 by one sample *t* test.

**c** Knock-down efficiency of AP2M1 in A549 cells transfected with sgRNAs (two sequences targeting AP2M1 and two non-targeting, NT) and non-transfected, was analyzed by Western blot. Representative blots and images are shown.

**
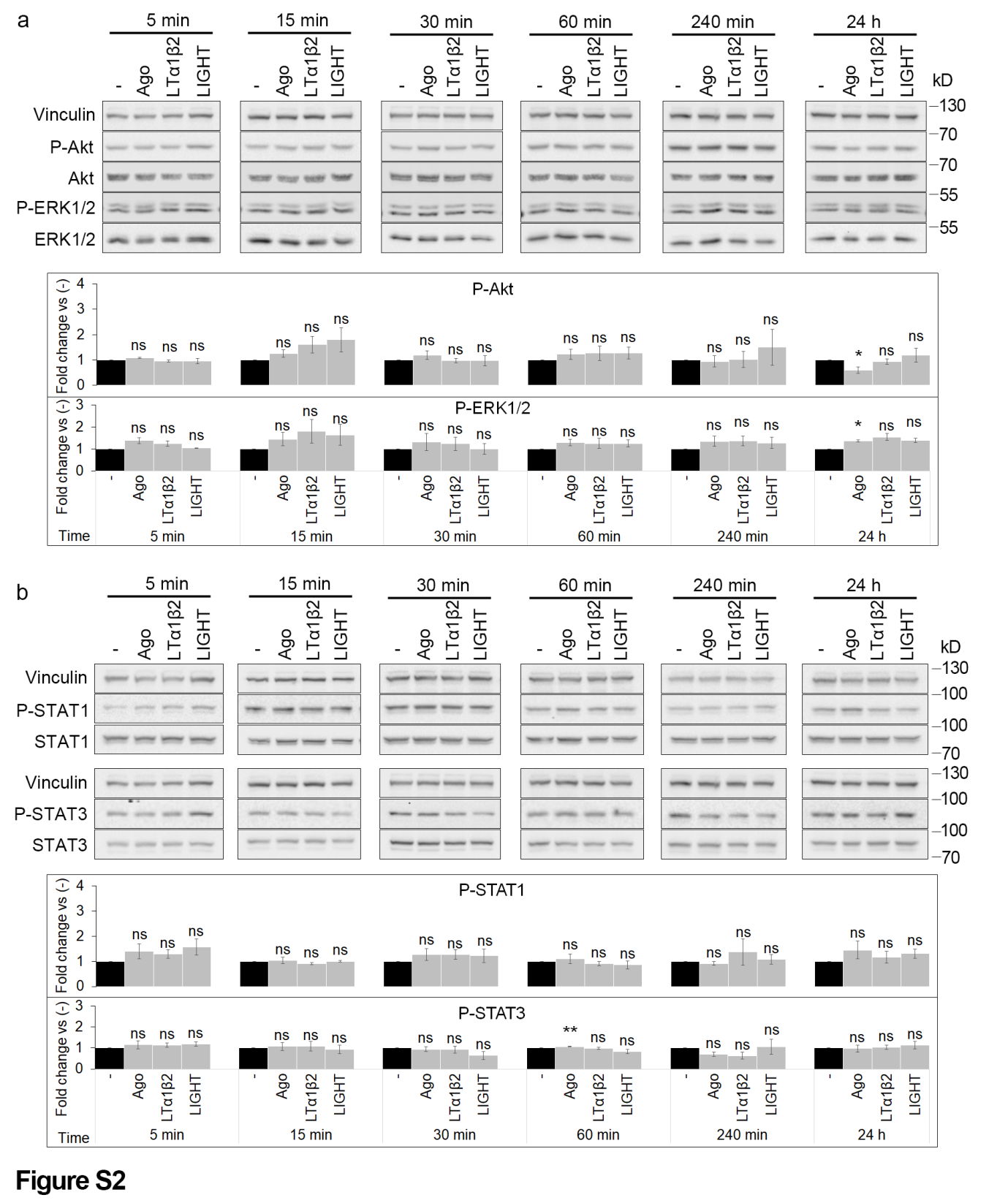
**

**Figure S2 Stimulation with LTβR ligands does not activate Akt, ERK1/2, STAT1 or STAT3.**

Lysates of A549 cells stimulated with: Ago, LTα1β2 and LIGHT for different time periods were analyzed by Western blotting with antibodies against the indicated proteins to assess activity of Akt and ERK1/2 (**a**), and STAT1 and STAT3 (**b**). P-Akt - phospho-Akt; P-ERK1/2 - phospho-ERK1/2; P-STAT1 - phospho-STAT1; P-STAT3 - phospho-STAT3. The blots of vinculin (loading control) in a are also used in lower part of panel b. The blots of vinculin in the upper part of panel b are also shown in Fig. 5b. Graphs show densitometric analysis of the abundance of the indicated proteins, normalized to loading control. Values are presented as a fold change vs unstimulated control (-), set as 1. Data represent the means ± SEM, n≥3; ns - P>0.05; *P≤0.05; **P≤0.01 by one sample *t* test. Representative blots are shown.

**
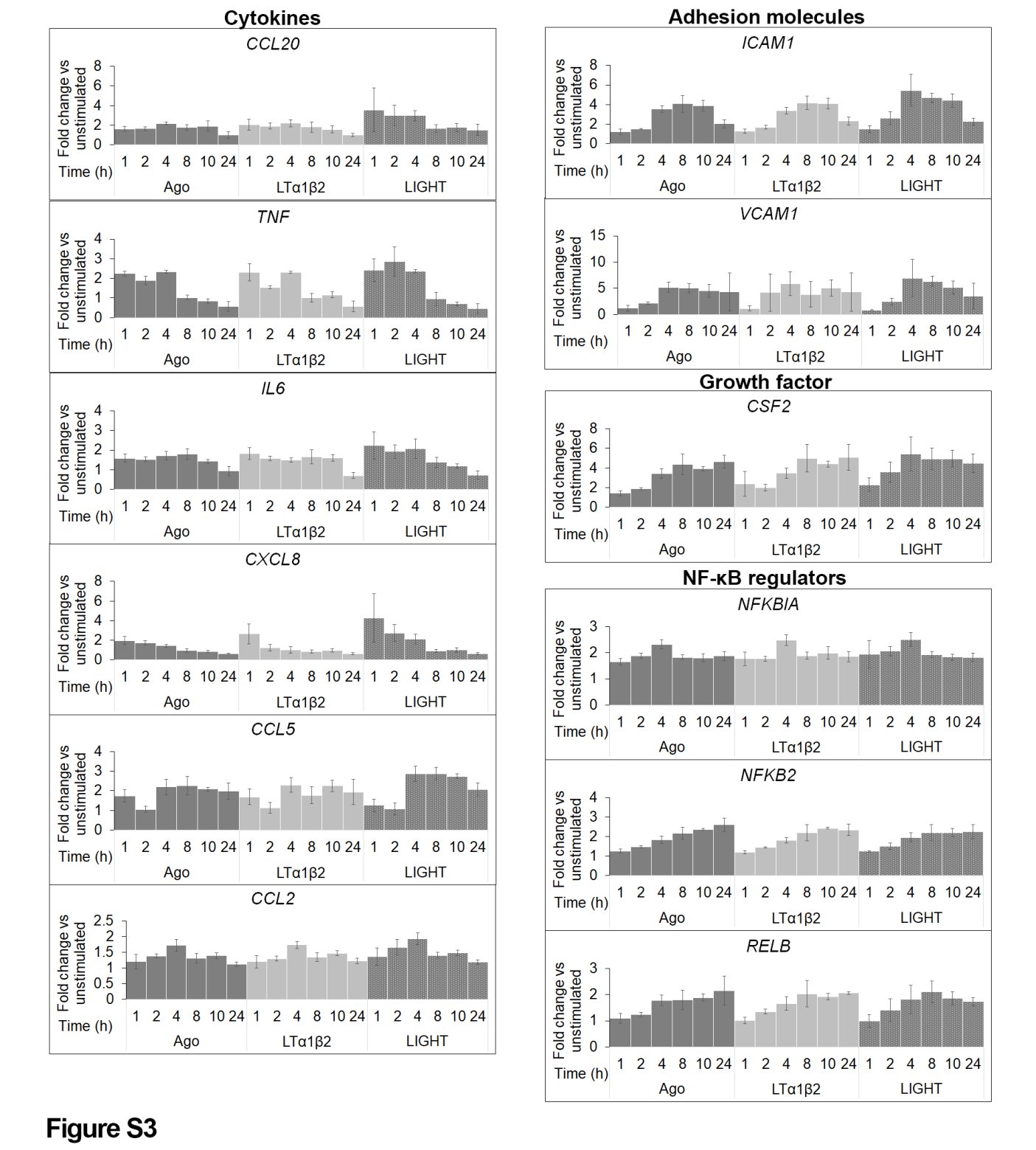
**

**Figure S3 Stimulation with LTβR ligands leads to expression of NF-κB target genes.**

mRNA levels of NF-κB target genes were analyzed by qRT-PCR in A549 cells stimulated with: Ago, LTα1β2 and LIGHT for different time periods. Values are presented as a fold change vs unstimulated cells. Data represent the means ± SEM, n=3.


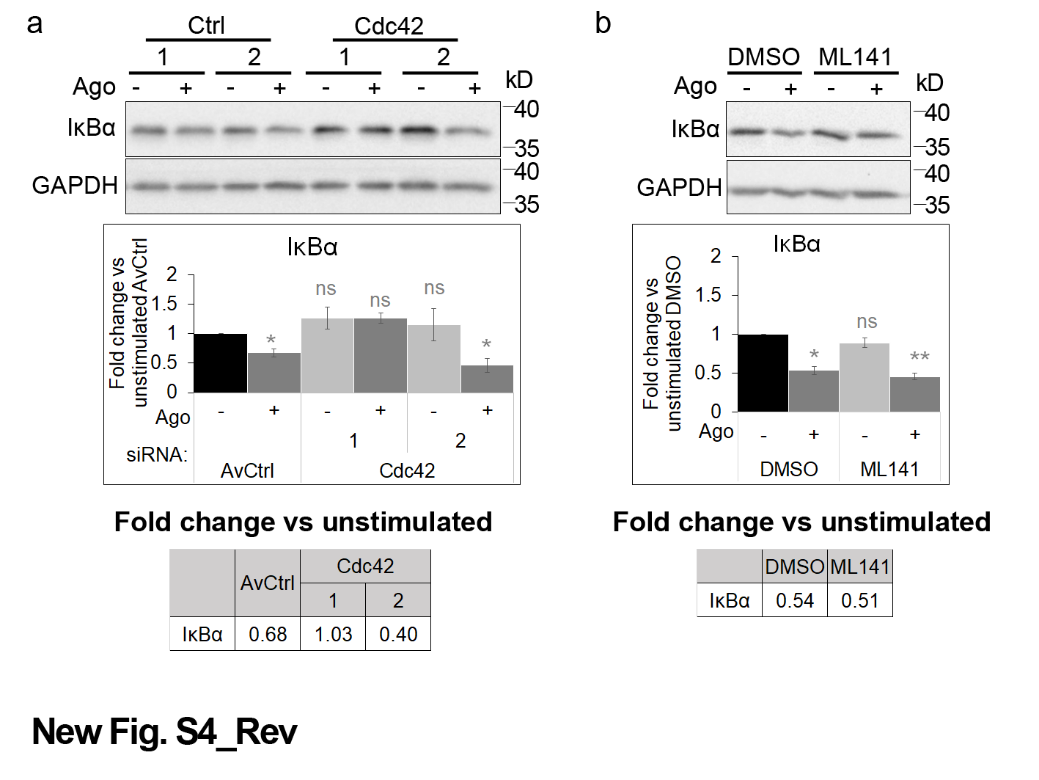


**Figure S4 Cdc42 deficiency does not affect the activation of canonical NF-κB signaling by LTβR.**

A549 cells were: transfected with siRNAs targeting Cdc42 (two oligonucleotides) (**a**) or treated with ML141 (**b**), along with the relevant controls, non-targeting siRNAs (two oligonucleotides, Ctrl) (a) or DMSO (b), and stimulated or not with Ago for 1 h. Lysates of cells were analyzed by Western blotting with antibodies against the indicated proteins. Representative blots are shown. Graphs show densitometric analysis of abundance of IκBα, normalized to loading control (GAPDH). Values are presented as a fold change vs unstimulated non-targeting controls – averaged non-targeting controls (AvCtrl) or DMSO, set as 1. Data represent the means ± SEM, n=4 (a), n=3 (b); ns - P>0.05; *P≤0.05; **P≤0.01 by one sample *t* test. Tables present the fold change of IκBα abundance in stimulated vs unstimulated cells (means, n≥3).


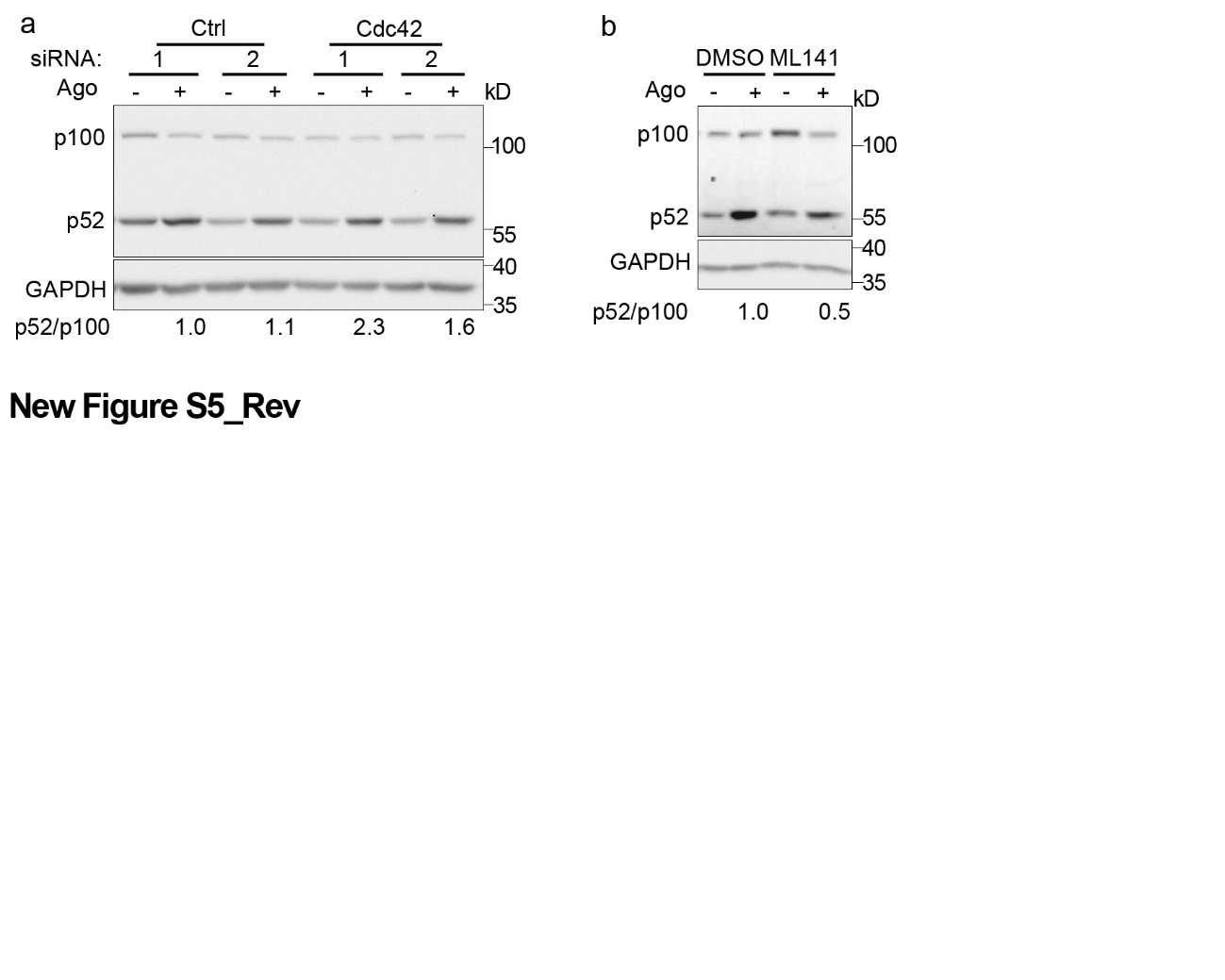


**Figure S5 Cdc42 deficiency does not affect the activation of non-canonical NF-κB signaling by LTβR.**

A549 cells were: transfected with siRNAs targeting Cdc42 (two oligonucleotides) (**a**) or treated with ML141 (**b**), along with the relevant controls, non-targeting siRNAs (two oligonucleotides, Ctrl) (a) or DMSO (b), and stimulated or not with Ago for 24 h. Lysates of cells were analyzed by Western blotting with antibodies against the indicated proteins. Representative blots are shown. Values presented below blots represent the averaged p52/p100/loading control ratio from at least three experiments (normalized to GAPDH, set as 1) in cells stimulated with Ago.

**
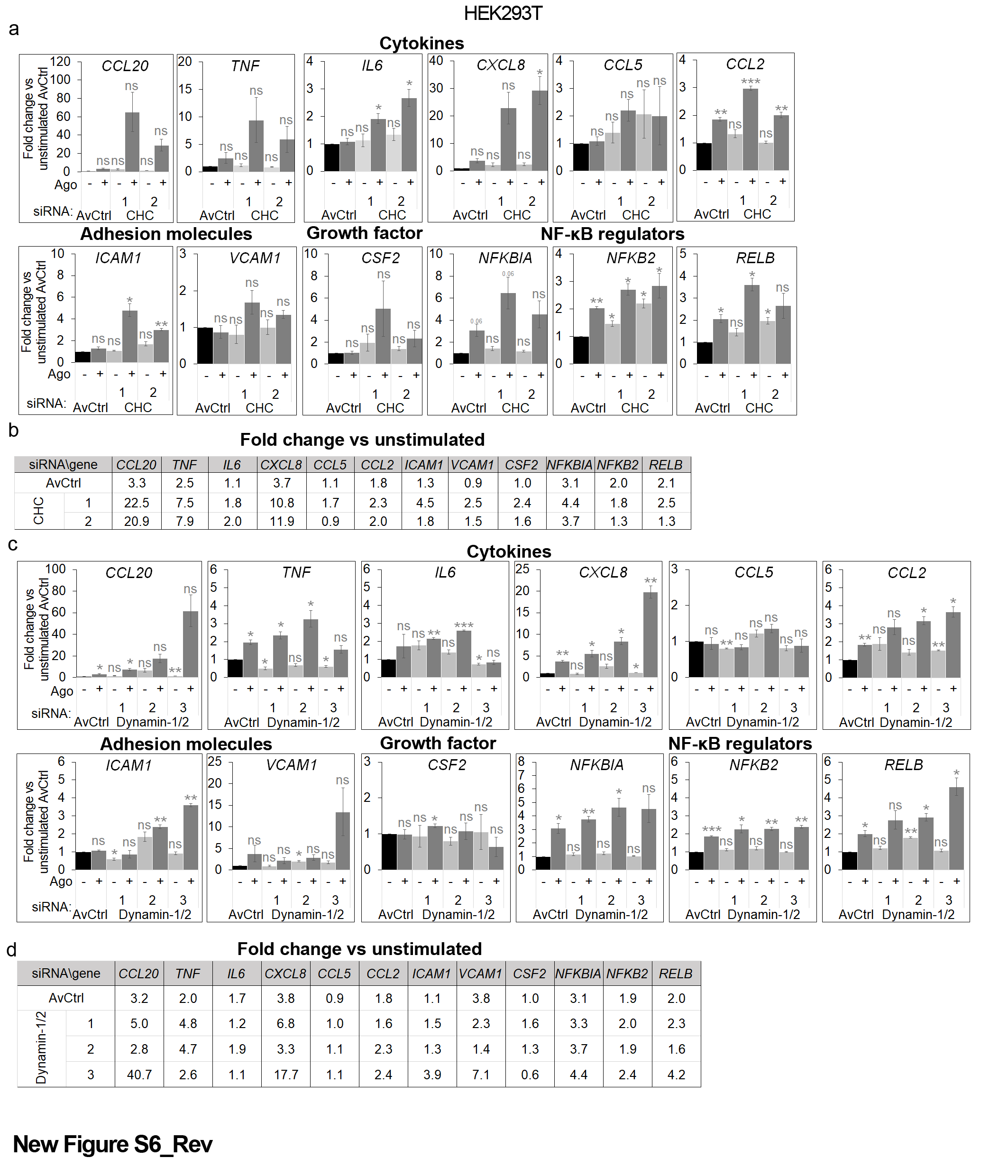
**

**Figure S6 Depletion of clathrin and dynamin enhances expression of LTβR target genes in HEK293T cells.**

mRNA levels of NF-κB target genes were analyzed by qRT-PCR in HEK293T cells transfected with siRNAs targeting clathrin (CHC, two oligonucleotides denoted with consecutive numbers) (**a**, **b**), dynamin-1/2 (three combinations of oligonucleotides targeting dynamin-1 and dynamin-2, see Methods) (**c**, **d**) and with relevant non-targeting siRNAs and stimulated with Ago for 2 h. Values are presented as a fold change vs unstimulated averaged non-targeting controls (AvCtrl), set as 1. Data represent the means ± SEM, n=3; ns - P>0.05; *P≤0.05; **P≤0.01; ***P≤0.001 by one sample *t* test. Tables present the fold change of expression of the indicated genes in stimulated vs unstimulated cells transfected with different combinations of siRNAs, targeting clathrin (b) and dynamin-1/2 (d), and non-targeting controls.


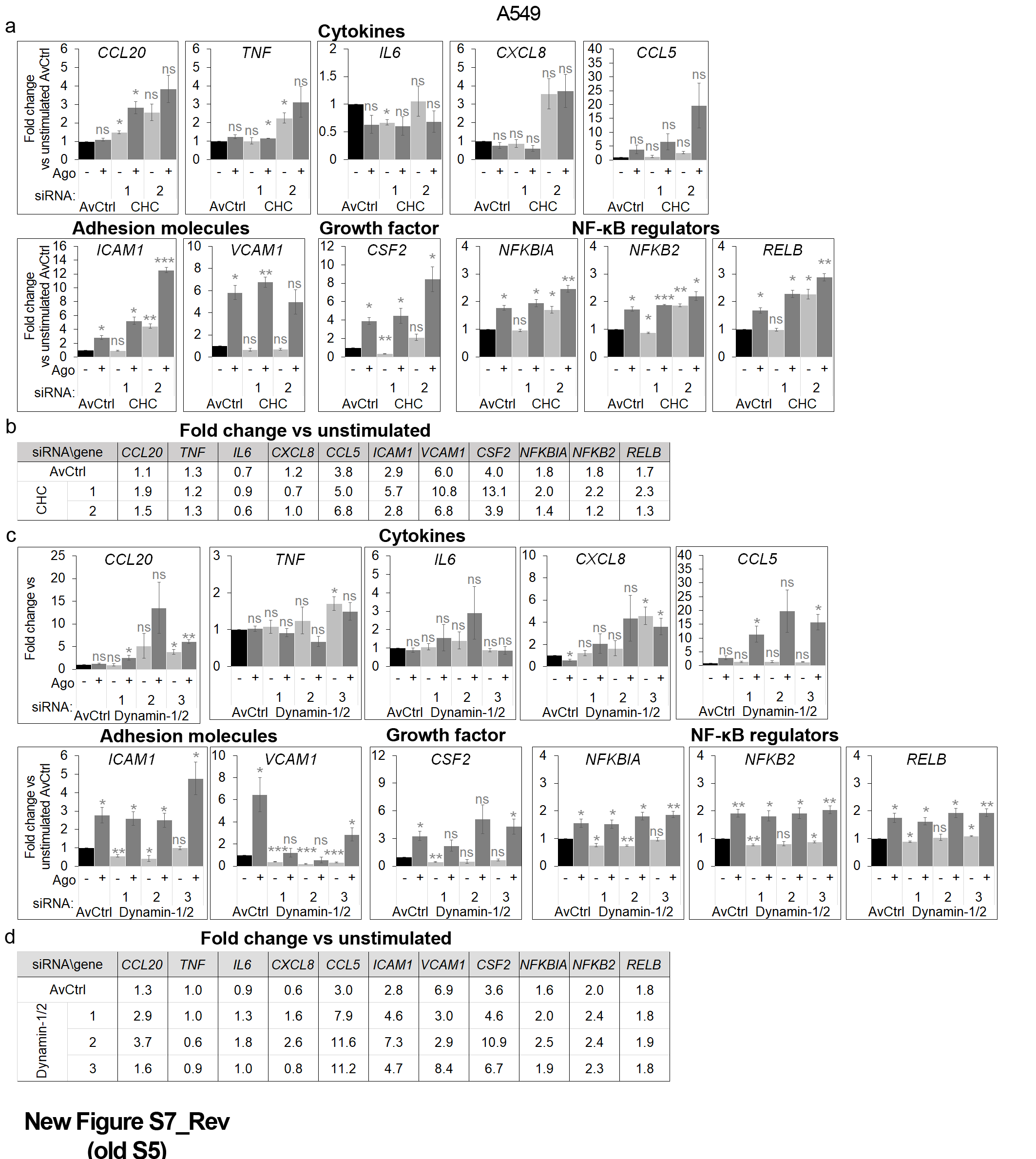


**Figure S7 Depletion of clathrin and dynamin enhances expression of the selected LTβR target genes in A549 cells upon prolonged stimulation.**

mRNA levels of NF-κB target genes were analyzed by qRT-PCR in A549 cells transfected with siRNAs targeting clathrin (CHC) (two oligonucleotides) (**a**, **b**) or dynamin-1/2 (three combinations of oligonucleotides targeting dynamin-1 and dynamin-2, see Methods) (**c**, **d**) and stimulated with Ago for 24 h. Values are presented as a fold change vs unstimulated averaged non-targeting controls (AvCtrl), set as 1. Data represent the means ± SEM, n=3; ns - P>0.05; *P≤0.05; **P≤0.01; ***P≤0.001 by one sample *t* test. Tables present the fold change of the indicated gene expression in stimulated vs unstimulated cells transfected with siRNAs, targeting clathrin (b) and dynamin-1/2 (d) and non-targeting controls.
